# Effects of Intermittent Hypoxia–Hyperoxia on Performance- and Health-Related Outcomes in Humans: A Systematic Review

**DOI:** 10.1186/s40798-022-00450-x

**Published:** 2022-05-31

**Authors:** Tom Behrendt, Robert Bielitzki, Martin Behrens, Fabian Herold, Lutz Schega

**Affiliations:** 1 Department of Sport Science, Chair for Health and Physical Activity, Otto-von-Guericke University Magdeburg, Universitätsplatz 2, 39104 Magdeburg, Germany; 2 Department of Orthopaedics, Rostock University Medical Center, Doberaner Str. 142, 18057 Rostock, Germany; 3 Research Group Degenerative and Chronic Disease, Movement, Faculty of Health Sciences, University of Potsdam, Karl-Liebknecht-Str. 24-25, 14476 Potsdam, Germany

**Keywords:** Hypoxic conditioning, Cognitive impairment, Metabolic disease, Cardiovascular disease, Geriatrics, Therapy

## Abstract

**Background:**

Intermittent hypoxia applied at rest or in combination with exercise promotes multiple beneficial adaptations with regard to performance and health in humans. It was hypothesized that replacing normoxia by moderate hyperoxia can increase the adaptive response to the intermittent hypoxic stimulus.

**Objective:**

Our objective was to systematically review the current state of the literature on the effects of chronic intermittent hypoxia–hyperoxia (IHH) on performance- and health-related outcomes in humans.

**Methods:**

PubMed, Web of Science™, Scopus, and Cochrane Library databases were searched in accordance with PRISMA guidelines (January 2000 to September 2021) using the following inclusion criteria: (1) original research articles involving humans, (2) investigation of the chronic effect of IHH, (3) inclusion of a control group being not exposed to IHH, and (4) articles published in peer-reviewed journals written in English.

**Results:**

Of 1085 articles initially found, eight studies were included. IHH was solely performed at rest in different populations including geriatric patients (*n* = 1), older patients with cardiovascular (*n* = 3) and metabolic disease (*n* = 2) or cognitive impairment (*n* = 1), and young athletes with overtraining syndrome (*n* = 1). The included studies confirmed the beneficial effects of chronic exposure to IHH, showing improvements in exercise tolerance, peak oxygen uptake, and global cognitive functions, as well as lowered blood glucose levels. A trend was discernible that chronic exposure to IHH can trigger a reduction in systolic and diastolic blood pressure. The evidence of whether IHH exerts beneficial effects on blood lipid levels and haematological parameters is currently inconclusive. A meta-analysis was not possible because the reviewed studies had a considerable heterogeneity concerning the investigated populations and outcome parameters.

**Conclusion:**

Based on the published literature, it can be suggested that chronic exposure to IHH might be a promising non-pharmacological intervention strategy for improving peak oxygen consumption, exercise tolerance, and cognitive performance as well as reducing blood glucose levels, and systolic and diastolic blood pressure in older patients with cardiovascular and metabolic diseases or cognitive impairment. However, further randomized controlled trials with adequate sample sizes are needed to confirm and extend the evidence. This systematic review was registered on the international prospective register of systematic reviews (PROSPERO-ID: CRD42021281248) (https://www.crd.york.ac.uk/prospero/).

## Key Points

• Current evidence indicates that chronic exposure to intermittent hypoxic–hyperoxic periods at rest can be considered an efficient non-pharmacological intervention strategy to improve physical and cognitive performance and reduce cardiometabolic risk factors in older patients with cardiovascular and metabolic diseases or cognitive impairment, when an intervention with 3–5 sessions per week over 3–6 weeks is conducted.

• Although the optimal hypoxic and hyperoxic dose and mode of application (i.e. at rest or in combination with exercise) are still unknown, from the available literature it can be inferred that 4–8 cycles of hypoxic–hyperoxic periods with moderate intensity (i.e. inspired fraction of oxygen of 0.10–0.12 and 0.30–0.40, respectively) and durations of 2–6 or 1–4 min per single hypoxic and hyperoxic period, respectively, are safe and well tolerated in older and younger adults.

• Still, there is no strong evidence that intermittent exposure to hypoxic–hyperoxic periods is more efficient than intermittent exposure to hypoxic–normoxic periods to improve performance- and health-related outcomes or reduce the session duration by shortening the reoxygenation periods.

## Introduction

Intermittent hypoxia (IH) is traditionally characterized by periodic and alternating cycles of hypoxia and normoxia. With the development and widespread availability of devices inducing a systemic or local hypoxic environment (e.g. hypobaric chambers, hypoxia rooms and tents, hypoxicators, or pneumatic cuffs), the “live low-train high” approach has gained considerable popularity as an effective and efficient training modality for a variety of professional athletes [1–3] as well as a non-pharmacological approach for the prevention and therapy of patients with various diseases or healthy adults, respectively [4, 5].

**Fig. 1:**
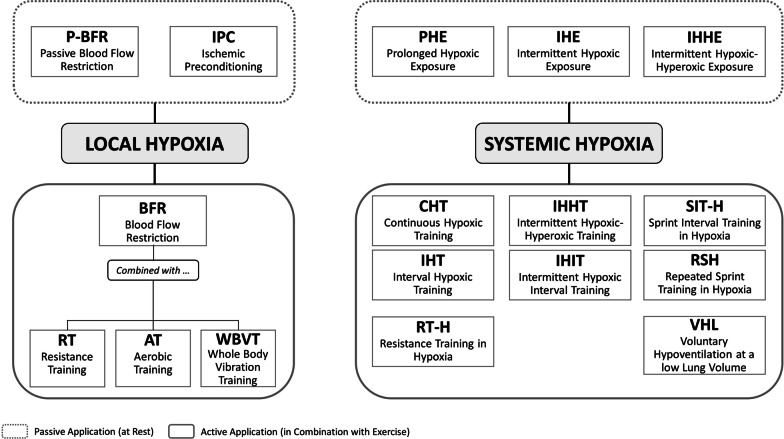
Graphical panorama of different “live low-train high” methods (modified from Girard et al. [2]). Please note that in the current literature the term “intermittent hypoxic–hyperoxic training” (IHHT) is commonly used for both passive and active applications. To avoid terminological ambiguity with respect to the term *intermittent hypoxic–hyperoxic periods*, we recommend to use the term "intermittent hypoxic–hyperoxic training" for active and “intermittent hypoxic–hyperoxic exposure” for passive applications

To date, different “live low-train high” methods exist (see Fig. 1). Commonly, systemic hypoxia can be generated in two ways: (1) by reducing the barometric pressure (BP, hypobaric hypoxia) or (2) by reducing the oxygen fraction in the inspired air (F_i_O_2_) via oxygen filtration or nitrogen dilution (normobaric hypoxia) [6]. Despite the ongoing debate whether different combinations of BP and F_i_O_2_ produce the same partial pressure of oxygen and trigger similar or different physiological responses [7–14], both types of hypoxia reduce arterial oxygen saturation (S_a_O_2_) [15, 16], which, in turn, stimulates specific biological signal cascades that promote hypoxia-induced adaptations. In particular, the reduction in S_a_O_2_ triggers the stabilization of hypoxia-inducible factors (HIF), which are the key oxygen sensors and master regulators of oxygen homeostasis regulating cellular adaptations to hypoxia [17, 18]. For example, the activation of the α-subunit of HIF (HIF-1α) upregulates genes that are responsible for erythropoiesis [19, 20], angiogenesis [20], and metabolic adaptations [21, 22] contributing to an increase in physical performance after long-term exposure to hypobaric and normobaric hypoxia [23]. From a practical point of view, inducing normobaric hypoxia is a more convenient, efficient, and less expensive form compared to hypobaric hypoxia [5], i.e. the creation of hypobaric hypoxia requires hypobaric chambers or expeditions to natural altitudes. As shown in Fig. 1, IH using normobaric hypoxia can be performed at rest or in combination with exercise, e.g. continuous or interval hypoxic training, (repeated) sprint interval training in hypoxia, or resistance training in hypoxia [2]. IH at rest refers to the use of either brief alternating hypoxic and normoxic periods (e.g. 3–6 min hypoxia and normoxia, respectively) of moderate- to relatively severe-intensity hypoxia (typically reported as F_i_O_2_ = 0.15–0.08, intermittent hypoxic exposure) or prolonged hypoxic exposures (0.5–4 h/session) at hypoxia intensities of F_i_O_2_ = 0.164–0.090 (prolonged hypoxic exposure) [4, 5, 24–26].

Studies involving normoxic control groups have revealed that neither intermittent nor prolonged hypoxic exposure could induce significant changes in haematological parameters or aerobic and anaerobic performance in elite athletes [1, 27–29]. Although IH at rest does not seem to improve sea-level performance of elite athletes, it might be a useful pre-acclimatization strategy for athletes or mountaineers before traveling to high altitudes [30–32]. However, high-intensity training under hypoxic conditions (e.g. repeated sprint training in hypoxia) [33–36] or a combination of hypoxic methods [1, 37] seems a promising approach for performance enhancement in moderately to well-trained populations and elite athletes. Nevertheless, it has also been stated that the use of hypoxic training methods (whether at rest or in combination with exercise) has been strongly promoted in elite athletes for many years without any evidence for their justification, which is still under debate [36, 38]. Studies conducted with healthy non-athletic populations have shown that IH at rest or in combination with physical exercises can be a valuable strategy to improve cognitive functions (e.g. selective attention and information processing speed [39, 40]) and health-related outcomes (e.g. vascular function [41] and glucose homeostasis [42]). Additionally, IH has been proposed as a promising non-pharmacological intervention for patients with, for example, cardiovascular, metabolic, and neurodegenerative diseases [43–47], as well as overweight and obese people [48]. In this context, studies have shown that intermittent hypoxic exposure improved aerobic capacity and exercise tolerance in elderly males with coronary artery disease [49] and reduced systolic and diastolic blood pressure in young adults with stage I hypertension [50]. Furthermore, prolonged hypoxic exposure performed over 22 days has been found to improve blood lipid profiles in patients with severe coronary artery disease [51] as well as aerobic capacity, skeletal muscle strength, quality of life, and left ventricular ejection fraction in patients with heart failure and reduced ejection fraction (≤ 35%) [52]. In addition, 3–8 weeks of intermittent hypoxic exposure also had positive effects in patients with prediabetes (i.e. reduction in fasting and 2 h post-oral blood glucose levels during a glucose tolerance test) [53], chronic obstructive pulmonary disease (i.e. increase in exercise tolerance, improved baroreflex sensitivity, and enhanced hypocapnic ventilatory response) [54, 55], and mild cognitive impairment (i.e. increase in cognitive functions and cerebral tissue oxygenation) [56]. Nevertheless, there is evidence that the combination of physical training (continuous cycling) and hypoxic exposure (continuous hypoxic training) provides some additional benefits compared to physical training in normoxia (i.e. a higher increase in peak oxygen consumption and maximal power output during cycling) in overweight and obese people [57].

In the last decade, a new IH-method was developed combining hypoxic and hyperoxic (F_i_O_2_ = 0.30–0.40) periods. Intermittent hypoxic–hyperoxic periods can be applied as a passive intervention modality with the subjects at rest (referred to as intermittent hypoxic–hyperoxic exposure, IHHE) or during physical exercise (referred to as intermittent hypoxic–hyperoxic training, IHHT). It has been hypothesized that replacing normoxia by moderate hyperoxia can increase the adaptive response to the intermittent hypoxic stimulus by upregulating reactive oxygen species (ROS) [58] and hypoxia-inducible genes [59]. While HIF-1α is stabilized when cellular oxygen content decreases [17], ROS is generated in the initial period of reoxygenation [60]. Although the excess of ROS is associated with cell damage and the pathogenesis of various diseases, a moderate ROS formation is also linked to beneficial physiological processes including (1) oxidation of damaged molecules, (2) synthesis of messenger molecules, and (3) extra- and intracellular signalling [61]. In particular, ROS triggers intracellular redox signal cascades, which activate transcription factors such as nuclear factor erythroid 2-related factor 2 (Nrf2) and HIF-1α by inactivating Kelch-like ECH-associated protein 1 (Keap1) and prolyl hydroxylase (PHD), respectively [62]. These factors are known to induce the expression of antioxidant and anti-inflammatory genes, heat shock proteins (HSP), iron regulation proteins, repair enzymes, erythropoietin (EPO), vascular endothelial growth factor (VEGF), and glycolytic enzymes promoting cell survival, erythropoiesis, blood vessel formation, and maintaining adenosine triphosphate level [58, 61, 63]. Therefore, the production of protective proteins and those responsible for the adaptations might be increased by replacing normoxia by hyperoxia periods without the need to increase hypoxia intensity. Thus, the application of intermittent hypoxia–hyperoxia, either passive or in combination with physical exercise, seems to be a promising intervention strategy for various populations.

Recently, placebo-controlled trials examined the effects of IHHE and IHHT [64–66]. For instance, Serebrovska et al. [66] investigated the effects of IHHE, intermittent hypoxic exposure, and sham hypoxia on carbohydrate and lipid metabolism as well as hypoxia resistance in 55 prediabetic patients (5 sessions per week for 3 weeks). The authors observed the same positive effect for both IHHE and intermittent hypoxic exposure [66]. However, it was concluded that IHHE leads to a faster reoxygenation resulting in a shorter session duration compared to intermittent hypoxic exposure (IHHE: 4 cycles of 5 min hypoxia and 3 min hyperoxia, intermittent hypoxic exposure: 4 cycles of 5 min hypoxia and 5 min normoxia). Another study compared the acute responses to IHHT, continuous hypoxic training, and sham hypoxia during aerobic exercise consisting of 40 min of moderate cycling in overweight non-insulin-dependent type 2 diabetic patients [64]. The authors revealed that both IHHT and continuous hypoxic training induced a greater up-regulation of pro-angiogenetic factors (e.g. VEGF and matrix metalloproteinase-9) than the sham hypoxia aerobic training without significant differences between the hypoxic modalities [64]. However, the authors noted that exercising under hypoxia–hyperoxia might be more tolerable than hypoxia–normoxia given the observed tendency for less exertion in IHHT (i.e. assessed with Borg’s Rating of Perceived Exertion scale) [64]. Consequently, there is some preliminary evidence that exercising under intermittent hypoxic–hyperoxic conditions may be a convenient, efficient, and less demanding training strategy to achieve similar positive effects as seen after training in hypoxia or intermittent hypoxic–normoxic conditions. This might be relevant for specific populations characterized by a low exercise tolerance and fitness level (e.g. sedentary individuals or patients with cardiovascular diseases).

Conclusively, IH at rest or in combination with physical exercise has been shown to be an effective intervention strategy to induce beneficial adaptations in several body systems that can positively influence the performance and health status of elite athletic or non-athletic people with or without disease. In order to enhance the IH effectiveness, researchers hypothesized that normoxia should be replaced by moderate hyperoxia [58, 67]. Indeed, in some studies promising effects of IHHE and IHHT on different performance- and health-related outcomes have been observed in healthy and preclinical populations [64–66]. However, to the best of our knowledge, the literature on the effects of IHHE and IHHT have yet not been systematically reviewed and summarized. To advance research and practical application of IHHE and IHHT, a systematic review and critical discussion of the results as well as methodology of IHHE and IHHT studies are required. Therefore, the present systematic review aimed to provide an overview and critical discussion of studies that have investigated the influence of IHHE and IHHT on performance- and health-related outcomes in humans.

## Methods

### Search Strategy and Process

This systematic review was conducted in accordance with the PRISMA guidelines (Preferred Reporting Items for Systematic Reviews and Meta-Analyses) [68, 69]. Two independent researchers (T.B. and F.H.) performed a systematic literature search in the following electronic databases [applied specifications/filters]: (1) PubMed [all fields/non]; (2) Scopus [all fields/source type: journal, document type: article]; (3) Web of Science [all fields/non]; and (4) Cochrane Library [all text/non]. The literature search included studies published from January 2000 to September 2021. To optimize the identification of relevant articles, the terms were combined with Boolean operators (“OR” and “NOT”). Terms combined with “NOT” were only searched for in the title and abstract.

To identify relevant articles, we used the following search terms in all electronic databases mentioned above:

$$\begin{array}{*{20}c} {\text{hypoxia}}{-}{\text{hyperoxia OR hyperoxia}}{-}{\text{hypoxia OR hypoxic OR hyperoxic}}{-} \\ {\text{hypoxic OR hypoxia/hyperoxia OR hyperoxia/hypoxia}} \\ {{\mathbf{NOT}}} \\ {\text{sleep apnoea OR sleep apnea OR neonates OR mice OR rats OR rabbits OR zebrafish}} \\ {\text{OR dog}} \\ \end{array}$$

Furthermore, references of the included studies (cross references) were checked for further potential articles. Any disagreements between the literature searchers were resolved through discussion and agreement.

**Fig. 2:**
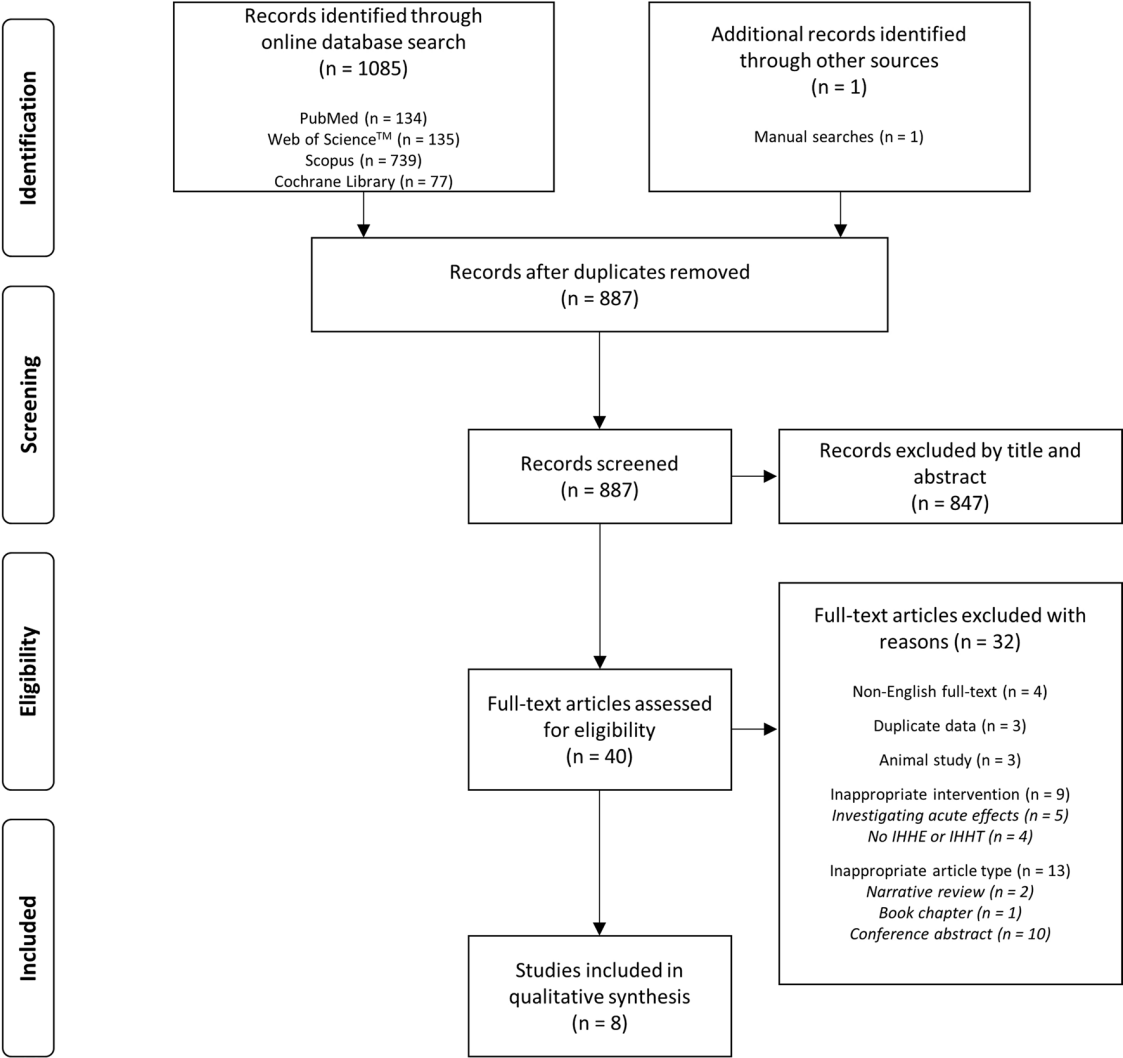
Flow chart of study selection. Please note that the term “inappropriate” refers to the inclusion and exclusion criteria used in this systematic review

The results of the systematic literature search were imported into a reference manager (Citavi 6.8, Swiss Academic Software GmbH, Switzerland) to analyse the retrieved studies (e.g. to remove duplicates, screen for relevant studies). The procedure is displayed in the flow chart shown in Fig. 2.

### In- and Exclusion Criteria

As recommended by the PRISMA guidelines [68, 69], we used the PICOS-principle [70] to define the eligibility criteria (i.e. specific exclusion and inclusion) for relevant studies. The inclusion and exclusion criteria are listed below.

#### Participants

We included all studies regardless of the sex and health status of the participants. Studies that have included participants with an age < 18 years or investigated animals were excluded.

#### Intervention

We included only studies that investigated the chronic effects of IHHE or IHHT on human performance or health. Thus, IHHE or IHHT had to be conducted regularly in a planned, structured, and purposed manner with the objective to affect one or multiple fitness or health dimensions. Studies that have investigated (1) the effects of acute IHHE or IHHT (i.e. a single IHHE or IHHT session), (2) only the effects of intermittent normoxia–hypoxia (i.e. without an IHHE or IHHT condition), and (3) the effects of permanent or long-term stay in hypoxia (e.g. long-term stay in high mountain regions) were excluded.

#### Comparison

We included all studies that involved a control group that was not exposed to IHHE or IHHT (e.g. placebo/sham control group).

#### Outcomes

We included all studies that assessed at least one or multiple performance- or health-related outcome(s).

#### Study Design

We included all longitudinal intervention studies that complied with the above-stated inclusion criteria and were published in English in a peer-reviewed scientific journal.

### Data Extraction

We extracted the following information from the included studies: (1) bibliographic information (first author and year of publication), (2) design information (study design and comparison group), (3) participants’ characteristics (health status, sex, age, body height, body mass, and body mass index), (4) characteristics of any additional exercise program if applicable (type and description of exercise, single session duration, training duration, training frequency, training density, and training setting), (5) characteristics of the IHHE or IHHT (hypoxia intensity, intra-session frequency [number of cycles], intra-session density [duration of a single hypoxic/hyperoxic period], total time of a single session, participants’ mean S_p_O_2_ at hypoxic condition, intervention duration, inter-session frequency of the intervention sessions, inter-session density of the intervention sessions, and number of total sessions across the intervention duration), and (6) main outcomes.

### Check for Duplicate Publication

To check for duplicate publication, we analysed each study using the decision tree for identification of patterns of duplicate publication by von Elm et al. [71]. The two criteria were similarity of study samples and similarity of study outcomes. Four duplicate patterns were defined: (1) pattern one = identical samples and identical outcomes, (2) pattern two = identical samples and different outcomes, (3) pattern three = different samples and identical outcomes, and (4) pattern four = different samples and different outcomes [71]. Studies matching one of these combinations were excluded from this systematic review. Three studies [72–74] were identified as duplicate category *pattern three* and were thus excluded from the final analysis (Fig. 2).

### Risk of Bias Assessment

Risk of bias assessment of the included studies was performed with the modified version of the Downs and Black checklist [75] used to assess the methodological quality of randomized controlled as well as non-randomized studies taking various aspects of the study design into account, e.g. reporting (Items 1–10), external validity (Items 11–13), internal validity (Items 14–26), and statistical power (Item 27). Given the specificity of studies investigating the chronic effect of IH, the importance of the hypoxic dose [25, 76], and the individual internal response to a hypoxic stimulus [77], we modified the checklist by adjusting Item 4 (description of the intensity of hypoxia and hyperoxia, number of hypoxic and hyperoxic periods per session [intra-session frequency], duration of hypoxic and hyperoxic periods [intra-session density]), Item 23 (homogeneity in main outcomes between groups at post-test), and by adding a further Item (Item 28: reporting of internal intensity of hypoxia [e.g. S_p_O_2_]). Each Item, except Item 5, was scored with one point if the criterion was met and with zero points if the criterion was not satisfied or could not be determined. Item 5 was scored with two points if all main confounders (i.e. sex, age, disability, training status, and body mass) were described, with one point if four of the five main confounders plus one secondary confounder (i.e. the moment of testing during the intervention or test mode) were described and with zero points if the described criterion was not met or was not appropriately acknowledged. Studies were classified based on the sum score as being of “good quality” (21–29 points), “moderate quality” (11–20 points), and “poor quality” (< 11 points) [78]. Three researchers (T.B., R.B., and M.B.) independently evaluated the risk of bias of the included studies and any case of disagreement in the ratings was resolved by discussion or consultation with a fourth author (F.H.).

## Results

### Study Selection

The systematic literature search revealed 1085 potentially relevant articles. One additional study was identified through the manual search of secondary data sources. After duplicates were removed, 887 studies remained and were assessed in the initial screening process. Of these 887 studies, titles or abstracts were screened, which resulted in the exclusion of 847 studies. Thus, 40 studies were examined for eligibility. Of these, 32 studies were excluded due to the following reasons: non-English full-text [79–82], duplicate data [72–74], investigating effects of IHHE or IHHT in animals [83–85], or did not meet the inclusion criteria with respect to the intervention (investigating only acute effects of IHHE or IHHT [64, 65, 86–88] or the effects of permanent or long-term stay in hypoxia or hyperoxia [89–92]), or the article type (i.e. not original article: narrative review [93, 94], book chapter [95], or a conference abstract [96–105]). After the full-text assessment, eight studies [66, 106–112] met our inclusion criteria and were qualitatively analysed. The study selection process is shown in Fig. 2. A meta-analysis was not possible because the included studies had a considerable heterogeneity concerning the investigated populations and outcome parameters.

### Risk of Bias Assessment

The average quality of the studies included in the qualitative analysis was rated as moderate. The median quality rating score on the modified Downs and Black checklist was 19 of the possible 29 points (range 17–22). Five studies [107–111] were rated as being of moderate quality, whereas the other three studies [66, 106, 112] were considered to be of good quality (Table 1). All studies scored zero points (i.e. the criterion was not satisfied or unable to determine) for Item 15 (blinding those measuring the main outcomes), Item 19 (reporting participants’ compliance with the intervention), Item 22 (recruiting participants over the same period of time), Item 24 (concealing randomized intervention assignment from patients and health care staff), and Item 28 (S_p_O_2_ values during hypoxia periods). Item 27 (sample size calculation) [109] was satisfied in only one of the eight studies.

**Table 1:** Results of risk of bias assessment using the modified checklist by Downs and Black [76]

References	Reporting										External validity			Internal validity						
	1	2	3	4	5	6	7	8	9	10	11	12	13	14	15	16	17	18	19	20
Bayer et al. [106]	1	1	1	1	1	1	1	1	1	1	1	1	1	1	0	1	1	1	0	1
Glazachev et al. [107]	1	1	1	1	2	1	1	1	1	0	1	1	1	1	0	1	1	1	0	0
Susta et al. [108]	1	1	1	1	2	1	1	0	1	1	1	1	1	1	0	1	1	1	0	1
Dudnik et al. [109]	1	0	1	1	0	1	1	1	1	1	1	1	1	1	0	1	1	1	0	0
Glazachev et al. [110]	1	1	1	1	0	1	1	1	0	1	1	1	0	1	0	1	1	1	0	1
Serebrovska et al. [66]	1	1	1	1	1	1	1	1	1	1	1	1	1	1	0	1	1	1	0	1
Serebrovska et al. [111]	1	1	1	1	1	1	1	0	0	1	1	1	1	1	0	1	1	1	0	1
Bestavashvili et al. [112]	1	1	1	1	1	1	1	1	1	1	1	1	1	1	0	1	1	1	0	1

References	Internal validity—confounder						Power	Hypoxia intensity	Total score
	21	22	23	24	25	26	27	28	∑
Bayer et al. [106]	1	0	1	0	1	1	0	0	22
Glazachev et al. [107]	1	0	0	0	0	1	0	0	19
Susta et al. [108]	0	0	0	0	0	1	0	0	19
Dudnik et al. [109]	1	0	1	0	0	1	1	0	19
Glazachev et al. [110]	1	0	1	0	0	0	0	0	17
Serebrovska et al. [66]	1	0	1	0	0	1	0	0	21
Serebrovska et al. [111]	1	0	1	0	0	1	0	0	19
Bestavashvili et al. [112]	1	0	1	0	1	1	0	0	22

### Participants’ Characteristics and Study Designs

All reviewed studies [66, 106–112] used IHHE. IHHE was performed in different populations, including geriatric patients [106], older patients with coronary arterial disease [107, 110], young track and field athletes with overtraining syndrome [108], older cardiology outpatients [109], older patients with prediabetes [66], older patients with mild cognitive impairment [111], and patients with metabolic syndrome [112]. Detailed information about the number of participants, sex distribution, and participants’ characteristics (e.g. age, height, weight, and body mass index) is provided in Table 2.

**Table 2:** Summary of study designs, participants’ characteristics, and characteristics of the interventions of the reviewed studies

References	Design	Participants	Training characteristics	Characteristics of IHHE
	(1) Study design (2) Comparison groups	(1) Participants’ characteristics (2) Number of participants (f/m) (3) Mean age ± SD in years (4) Mean height ± SD in cm/mean weight ± SD in kg/mean BMI ± SD in kg/m^2^	(1) Type and description of exercise (2) Single session duration (3) Training duration (4) Training frequency (5) Training density (6) Training setting	(1) Intensity of hypoxia/hyperoxia (F_i_O_2_) (2) Intra-session frequency (number of cycles) (3) Intra-session density (Duration of a single hypoxic/hyperoxic period) (4) Total time of IHHE procedure (5) Participants’ mean S_p_O_2_ at IHHE (hypoxic condition) (6) Intervention duration (7) Inter-session frequency of IHHE sessions (8) Inter-session density of IHHE sessions (9) Number of total sessions across the intervention duration
Bayer et al. [106]	(1) Randomized controlled trial (2) 2 groups [1] IHHE (normobaric IHHE and individual multimodal rehabilitation training) [2] Sham IHHE (simulated IHHE (normobaric normoxic air) and individual multimodal rehabilitation training)	(1) Geriatric patients (2) IHHE: 18 (13/5) Sham IHHE: 16 (14/2) (3) IHHE: 80.9 ± 7.8 Sham IHHE: 83.4 ± 5.5 (4) IHHE: 163.7 ± 8.3/72.0 ± 9.3/27.0 ± 3.9 Sham IHHE: 163.2 ± 8.5/66.8 ± 12.3/25.0 ± 6.6	(1) Individual multimodal training^a^ (2) N.R. (3) 5–6 weeks (4) 2–3 sessions/week (16–20 sessions) (5) N.R. (6) 30 min physiotherapy (balance training, coordination training, and exercises to stimulate energy metabolism), 60 min occupational therapy (motor functional training, perceptual training, mental training, and cognitive training), and 20 min cycling	(1) 0.12/0.35 (2) N.R. (3) 4–6 min/1–2 min (4) 35–45 min (5) N.R. (6) 5–6 weeks (7) 2–3 sessions/week (8) N.R. (9) 14–15 sessions
Glazachev et al. [107 ]	(1) Controlled trial (2) 2 groups [1] IHHE (normobaric IHHE) [2] Sham IHHE (patients were enrolled after completing a standard cardiac rehabilitation program (8 weeks, 2 days/week), simulated IHHE (normobaric normoxic air))	(1) Patients with coronary arterial disease (NYHA functional class II and III) (2) IHHE: 27 (18/9) Sham IHHE: 19 (10/9) (3) IHHE: 63.9 ± 13.9 Sham IHHE: 79.1 ± 12.5 (4) IHHE: N.R./81.6 ± 13.9/N.R. Sham IHHE: N.R./79.1 ± 12.5/N.R.	(1) N.A. (2) N.A. (3) N.A. (4) N.A. (5) N.A. (6) N.A.	(1) 0.10–0.12/0.30–0.35 (2) 5–7 cycles (3) 4–6 min/3 min (4) N.R. (5) N.R. (6) 5 weeks (7) 3 sessions/week (8) N.R. (9) 15 sessions
Susta et al. [108]	(1) Pilot study (2) 2 groups [1] IHHE (normobaric IHHE and low-intensity running performed by athletes with overtraining syndrome) [2] Control group (healthy athletes performing training as usual)	(1) Young track and field athletes with and without overtraining syndrome (2) IHHE: 15 (8/7) CG: 19 (12/7) (3) Overall: 18–20 (4) Overall: 176.4 ± 14.6/71.4 ± 6.9/N.R.	(1) 2 bouts of 30 min running at 40% VO_2max_ with 10 min rest^a^ (2) 70 min (3) 4 weeks (4) 3 days/week (5) N.R. (6) Low-intensity running	(1) 0.11/0.30 (2) 6–8 cycles (3) 5–7 min/2–6 min (4) 40–50 min (5) N.R. (6) 4 weeks (7) 3 sessions/week (8) N.R. (9) 12 sessions
Dudnik et al. [109]	(1) Randomized controlled trial (2) 2 groups [1] IHHE (normobaric IHHE) [2] Sham IHHE (simulated IHHE (normobaric normoxic air) and exercise program)	(1) Cardiology outpatients (2) IHHE: 15 (N.R.) Sham IHHE: 14 (N.R.) (3) IHHE: 66.7 ± 5.7 Sham IHHE: 65.0 ± 6.2 (4) IHHE: N.R./N.R./27.7 ± 2.3 Sham IHHE: N.R./N.R./28.9 ± 2.0	(1) Standard tailored cardiopulmonary exercise program according to the European Society of Cardiology^b^ (2) N.R. (3) 8 weeks (4) 150 min/week (5) N.R. (6) 12–13 at Borg scale and/or 64–75% of maximal heart rate	(1) 0.11–0.12/0.30–0.33 (2) 5–7 cycles (3) 4–6 min/3 min (4) N.R. (5)
Glazachev et al. [110]	(1) Randomized controlled trial (2) 2 groups [1] IHHE (normobaric IHHE) [2] Sham IHHE (simulated IHHE (normobaric normoxic air))	(1) Patients with chronic coronary artery disease and angina pectoris of functional class II–III (2) Overall: 36 (26/10) IHHE: 17 (N.R.) Sham IHHE: 19 (N.R.) (3) Overall: 68.2 ± 6.1 (4) N.R./N.R./N.R.	(1) N.A. (2) N.A. (3) N.A. (4) N.A. (5) N.A. (6) N.A.	(1) 0.11–0.12/0.35 (2) N.R. (3) 2–6 min/1–2 min (4) 45–50 min (5) N.R. (6) 3 weeks (7) 5 sessions/week (8) 1 session per day for 5 days and 2 days rest (e.g. Monday to Friday: training, Saturday and Sunday: rest) (9) 15 sessions
Serebrovska et al. [66]	(1) Randomized controlled trial (2) 3 groups [1] IHHE (normobaric IHHE) [2] IHE (normobaric intermittent hypoxic exposure) [3] Sham IHHE (simulated IHHE (normobaric normoxic air))	(1) Patients with prediabetes (2) IHHE: 17 (13/4) IHE: 22 (15/7) Sham IHHE: 16 (10/6) (3) IHHE: 67.7 ± 7.7 IHE: 64.2 ± 6.6 Sham IHHE: 67.5 ± 8.7 (4) IHHE: 163 ± 6.0/84.9 ± 12.8/32.2 ± 4.6 IHE: 164 ± 9.5/86.3 ± 14.2/32.5 ± 6.7 Sham IHHE: 163 ± 6.0/84.9 ± 12.8/32.2 ± 4.6	(1) Intermittent hypoxic exposure^b^ (2) N.R. (3) 3 weeks (4) 5 sessions/week (15 sessions) (5) N.R. (6) Intermittent hypoxic exposure (5 min of hypoxia (12% F_i_O_2_) and 5 min of normoxia (~ 21% F_i_O_2_))	(1) 0.12/0.33 (2) 4 cycles (3) 5 min/3 min (4) N.R. (5) N.R. (lowest: ~ 79%) (6) 3 weeks (7) 5 sessions/week (8) N.R. (9) 15 sessions
Serebrovska et al. [111]	(1) Pilot study (2) 3 groups [1] IHHE (patients with mild cognitive impairments performing normobaric IHHE) [2] Sham IHHE (patients with mild cognitive impairments performing simulated IHHE (normobaric normoxic air)) [3] Control group (healthy participants performing either IHHE nor Sham IHHE)	(1) Patients with mild cognitive impairments (2) IHHE: 8 (6/7) Sham IHHE: 6 (6/0) Control group: 7 (6/1) (3) IHHE: 68.2 ± 7.2 Sham IHHE: 72.6 ± 6.9 Control group: 63.0 ± 10.0 (4) IHHE: N.R./N.R./27.7 ± 2.0 Sham IHHE: N.R./N.R./26.3 ± 5.5 Control group: N.R./N.R./26.5 ± 3.6	(1) N.A. (2) N.A. (3) N.A. (4) N.A. (5) N.A. (6) N.A.	(1) 0.12/0.33 (2) 4 cycles (3) 5 min/3 min (4) N.R. (5) N.R. (6) 3 weeks (7) 5 sessions/week (8) N.R. (9) 15 sessions
Bestavashvili et al. [112]	(1) Randomized controlled trial (2) 2 groups [1] IHHE (normobaric IHHE) [2] Sham IHHE (simulated IHHE (normobaric normoxic air))	(1) Patients with metabolic syndrome (2) IHHE: 32 (18/14) Sham IHHE 33 (14/19) (3) IHHE: 60.0 (45.5; 65.5) Sham IHHE: 61.5 (56.2; 66.0) (4) IHHE: N.R./92.0 (81.0; 114.0)/34.3 (30.2; 38.0) Sham IHHE: N.R./92.5 (82.8; 104.0)/32.4 (30.8; 35.8)	(1) N.A. (2) N.A. (3) N.A. (4) N.A. (5) N.A. (6) N.A.	(1) 0.11–0.12/0.30–0.35 (2) N.R. (3) 4–7 min/2–4 min (4) 40–45 min (5) N.R. (6) 3 weeks (7) 5 sessions/week (8) One session per day for 5 days and 2 days rest (e.g. Monday to Friday: training, Saturday and Sunday: rest) (9) 15 sessions

*BMI* body mass index, *CAD* coronary artery disease, *CG* control group, *f* female, *F*_*i*_*O*_*2*_ fraction of inspired oxygen, *IHE* intermittent hypoxic exposure, *IHHE* intermittent hypoxia–hyperoxia exposure, *m* male, *N.A.* not available, *N.R.* not reported, *NYHA* New York Heart Association, *RIP* remote ischaemic preconditioning, *SD* standard deviation, *S*_*P*_*O*_*2*_ blood oxygen saturation measured with finger pulse oximeter

^a^Describes the characteristics of an additional training that is carried out in addition to the IHHE

^b^Describes the characteristics of the training that is performed by an control group

Five studies [66, 106, 109, 110, 112] were classified as randomized controlled trials, one study [107] as a non-randomized controlled trial, and two studies [108, 111] were described as pilot studies. In seven studies [66, 106, 107, 109–112], the IHHE intervention group was compared to at least one control group performing a sham IHHE. One study [108] compared IHHE with a physically active healthy control group. Additionally, in some studies, IHHE was further compared with intermittent hypoxic exposure [66] as well as a physically active [109] or inactive control group [111]. In two studies, IHHE was performed in addition to an individualized multimodal training program (consisting of 30 min of physiotherapy procedures, 60 min of occupational therapy, and 20 min of aerobic training) [106] or low-intensity aerobic exercise (consisting of two bouts of 30 min running at 50% of maximum oxygen uptake, with 10 min rest between bouts) [108].

### Characteristics of the Intermittent Hypoxia–Hyperoxia Protocols

All studies used normobaric hypoxia and hyperoxia (Table 2). The hypoxic and hyperoxic gas mixture was administered via face masks connected to hypoxia generators. The intensity of hypoxia and hyperoxia ranged from F_i_O_2_ = 0.10–0.12 and F_i_O_2_ = 0.30–0.40, respectively. The mean S_p_O_2_ value of the patients during the hypoxia cycles was not reported in the studies. Five studies [66, 107–109, 111] reported the number of hypoxic–hyperoxic cycles per session. The number of cycles in these studies ranged from 4 to 8 cycles per session. The cycle duration for the hypoxia and hyperoxia periods ranged from 2 to 7 min and 1 to 6 min, respectively. Four studies [106, 108, 110, 112] reported the total time taken for a single IHHE procedure with a minimum of 35 and a maximum of 50 min. Based on the number of cycles and the duration of the hypoxic and hyperoxic periods, it can be assumed that the entire training session lasted approximately 35–63 min [107, 109] and 32 min [66, 111] in the studies not reporting the total duration. IHHE was performed with a frequency of 2–5 sessions per week, over an intervention period of 3–6 weeks (12–15 sessions in total) [66, 106–112]. The inter-session density of the IHHE intervention (i.e. distribution of IHHE sessions across a distinct time interval with regard to recovery time in-between the IHHE sessions) was reported in the study from Glazachev et al. [110] and Bestavashvili et al. [112] (5 weekly IHHE sessions and 2 days of rest per week). In seven studies, [66, 106–110, 112] the patients’ individual reaction to a hypoxic stimulus was determined with a hypoxia test that was conducted prior to the IHHE intervention. The hypoxia test consisted of breathing a hypoxic gas mixture (F_i_O_2_ = 0.10–0.12) for 10–20 min under constant monitoring of heart rate or S_p_O_2_ or both. Six studies [106–110, 112] stated that the IHHE protocol (i.e. duration or intensity of hypoxia and hyperoxia periods) was individually adjusted based on the results of the hypoxia test and the individual responses (heart rate and S_p_O_2_). Two studies [66, 111] used fixed parameters (i.e. hypoxia and hyperoxia intensity, inter-session density [i.e. cycle duration], inter-session frequency [i.e. number of cycles]).

### Effects of Intermittent Hypoxia–Hyperoxia on Physical and Cognitive Performance as well as Haematological, Metabolic, and Haemodynamic Parameters

The included studies investigated the effect of IHHE on different outcomes including physical [106–110] and cognitive performance [106, 111] as well as metabolic [66, 107, 110, 112], haemodynamic [106–109], and haematological parameters [107–109]. The main findings of the eight included studies are summarized in Table 3.

**Table 3:** Summary of assessed outcomes and main results of the reviewed studies

References	Assessed outcomes	Main results
Bayer et al. [106]	*Cognitive functions* Dementia detection test (DemTect) Clock drawing test (CDT) *Physical functions* Six-minute Walk Test (6MWT) *Cardiovascular hemodynamic parameters* Resting heart rate Resting systolic and diastolic blood pressure Resting oxygen saturation	*Within-group comparisons (pre-test vs. post-test)* ↑ DemTect in IHHE (11.2 ± 3.5 points vs. 14.2 ± 3.7 points) ↑ CDT in IHHE (7.8 ± 2.9 points vs. 8.4 ± 3.0 points) ↑ 6MWT in IHHE and sham IHHE (234.3 ± 94.7 m vs. 290.7 ± 83.1 m; 250.6 ± 94.3 m vs. 277.7 ± 96.3 m) *Between-group comparisons* ↑ DemTect in IHHE compared to sham IHHE (post-test: 14.2 ± 3.7 points vs. 11.3 ± 3.6 points) ↑ CDT in IHHE compared to sham IHHE (post-test: 8.4 ± 3.0 points vs. 6.8 ± 2.6 points) ↑ 6MWT in IHHE compared to sham IHHE (post-test: 290.7 ± 83.1 m vs. 277.7 ± 96.3 m) *Correlations* *Δ*-DemTect ↔ *Δ*-6MWT (*r* = + 0.57) *Δ*-CDT ↔ *Δ*-6MWT (*r* = + 0.42)
Glazachev et al. [107]	*Physical functions* Cardiopulmonary exercise test *Cardiovascular hemodynamic parameters* Resting heart rate Resting systolic and diastolic blood pressure Resting left ventricular ejection fraction *Blood markers* Haemoglobin concentration, reticulocytes, total cholesterol, high- and low-density lipoprotein, and glucose Atherogenic index ((total cholesterol − high-density lipoprotein) ÷ high-density lipoprotein) *Quality of life * Seattle Angina Questionnaire (SAQ)	*Within-group comparisons (pre-test vs. post-test vs. 1-month follow-up)* ↓ Angina as a reason to stop cardiopulmonary exercise test in IHHE (12 vs. 6 vs. 3^b^, ^c^) ↑ Time to exhaustion in cardiopulmonary exercise test (modified Bruce protocol) in IHHE (354 ± 194 s vs. 383 ± 141 s vs. 395 ± 130 s^b^) ↑ Time to exhaustion in cardiopulmonary exercise (Bruce protocol) in IHHE (280 ± 126 s vs. 295 ± 79 s vs. 332 ± 113 s^b^) ↑ VO_2peak_ in IHHE (14.3 ± 4.2 ml-O2/min/kg vs. 16.1 ± 4.2 ml-O2/min/kg^a^ vs. 15.4 ± 4.5 ml-O2/min/kg^a^) ↓ Systolic blood pressure in IHHE (151 ± 19 mmHg vs. 130 ± 13 mmHg^a^ vs. 129 ± 11 mmHg^b^) ↓ Diastolic blood pressure in IHHE (85 ± 11 mmHg vs. 73 ± 7 mmHg^a^ vs. 75 ± 9 mmHg^b^) ↓ Resting heart rate in IHHE (71.5 ± 11.4 beats/min vs. 67.7 ± 8.3 beats/min^a^ vs. 66.6 ± 10.0 beats/min^b^) ↓ Maximum heart rate in IHHE (122 ± 19 beats/min vs. 120 ± 14 beats/min^a^ vs. 116 ± 14 beats/min^b^) ↑ Left ventricle ejection fraction in IHHE (14.3 ± 4.2% vs. 16.1 ± 4.2%^a^ vs. 15.4 ± 4.5%^b^) ↑ Reticulocytes in IHHE (9.0 ± 4.5% vs. 11.3 ± 6.2%^a^ vs. 9.2 ± 4.8%^b^) ↓ Total cholesterol in IHHE (5.6 ± 1.4 mmol/L vs. 5.1 ± 1.2 mmol/L^a^ vs. 5.5 ± 1.4 mmol/L^b^) ↓ Low-density lipoprotein in IHHE (3.5 ± 1.2 mmol/L vs. 3.2 ± .9 mmol/L^a^ vs. 2.6 ± 1.3 mmol/L^b^, ^c^) ↓ Atherogenic index in IHHE (4.7 ± 1.8 vs. 3.4 ± 1.3^a^ vs. 3.5 ± 1.5^c^) ↓ Glucose in IHHE (7.1 ± 2.3 mmol/L vs. 6.5 ± 1.7 mmol/L vs. 6.2 ± 1.7 mmol/L^c^) ↑ SAQ physical limitation subscale in IHHE (43.3 ± 17.7 vs. 51.6 ± 13.1^a^ vs. 53.7 ± 17.8^b^) ↑ SAQ angina stability subscale in IHHE (56.5 ± 27.4 vs. 78.3 ± 23.3^a^ vs. 79.6 ± 22.7^b^) ↑ SAQ angina frequency subscale in IHHE (59.6 ± 27.6 vs. 81.1 ± 17.9^a^ vs.80.9 ± 18.2^b^) ↑ SAQ treatment satisfaction subscale in IHHE (60.7 ± 16.2 vs. 77.4 ± 16.8^a^ vs. 80.5 ± 17.7^b^) ↑ SAQ disease perception subscale in IHHE (47.2 ± 18.9 vs. 60.8 ± 17.8 vs. 63.4 ± 17.4^b^) *Between-group comparisons* ↓ Angina as a reason to stop cardiopulmonary exercise test in IHHE compared to sham IHHE (1-month follow-up: 3 vs. 6) ↑ Exercise time (modified Bruce protocol) in IHHE compared to sham IHHE (post-test: 383 ± 141 s vs. 280 ± 92) ↑ VO_2peak_ in IHHE compared to sham IHHE (1-month follow-up: 15.4 ± 4.5 ml-O_2_/min/kg vs. 17.8 ± 4.9 ml-O_2_/min/kg) ↑ Reticulocytes in IHHE compared to sham IHHE (post-test: 11.3 ± 6.2% vs. 6.4 ± 3.6%; 1-month follow-up: 9.2 ± 4.8% vs. 5.11 ± 3.13%) ↓ Total cholesterol in IHHE compared to sham IHHE (post-test 5.1 ± 1.2 mmol/L vs. 5.5 ± 0.9 mmol/L) ↓ Low-density lipoprotein in IHHE compared to sham IHHE (post-test: 3.2 ± .9 mmol/L vs. 3.6 ± 0.8 mmol/L; 1-month follow-up: 2.6 ± 1.3 mmol/L vs. 3.5 ± 0.8 mmol/L) ↓ Atherogenic index in IHHE compared to sham IHHE (post-test: 3.4 ± 1.3 vs. 3.6 ± 1.1) ↑ Atherogenic index in IHHE compared to sham IHHE (1-month follow-up: 3.5 ± 1.5 vs. 3.4 ± 1.0)
Susta et al. [108]	*Physical functions* Cardiopulmonary exercise test *Cardiovascular hemodynamic parameters* Inotropic reserve index (IRI, (maximal systolic blood pressure − resting systolic blood pressure) ÷ resting systolic blood pressure) Chronotropic reserve index (CRI, (maximal heart rate − resting heart rate) ÷ resting heart rate) Resting heart rate and heart rate variability *Cardiovascular hemodynamic parameters* Inotropic reserve index (IRI, (maximal systolic blood pressure − resting systolic blood pressure) ÷ resting systolic blood pressure) Chronotropic reserve index (CRI, (maximal heart rate − resting heart rate) ÷ resting heart rate) Resting heart rate and heart rate variability *Blood markers* Red blood cell count, reticulocyte, haemoglobin concentration, and haematocrit *Hypoxia test (10 min at F*_i_*O*_2_ *= 0.10)* Oxygen saturation (S_p_O_2_) Maximal heart rate (HR_max_)	*Within-group comparisons (pre-test vs. post-test)* ↑ PWC170 in IHHE (170.8 ± 44.8 W vs. 191.9 ± 26.9 W) ↓ IRI in IHHE (65.8 ± 3.6% vs. 54.8 ± 5.4%) ↓ CRI in IHHE (50.0 ± 5.3% vs. 38.0 ± 5.9%) ↑ S_p_O_2_ during hypoxic test in IHHE (77.9 ± 6.8% vs. 84.2 ± 5.7%) ↓ HR_max_ during hypoxic test in IHHE (82.2 ± 14.6 beats/min vs. 76.6 ± 11.0 beats/min) ↑ Standard deviation of R–R intervals in IHHE (54.0 ± 24.7 ms vs. 76.0.2 ± 26.8 ms) ↓ Low frequency power in IHHE (1300 ± 661 ms^2^ vs. 801 ± 673 ms^2^) ↑ High frequency power in IHHE (277 ± 188 ms^2^ vs. 624 ± 468 ms^2^) ↓ Low frequency to high frequency index in IHHE (8.01 ± 7.51 vs. 1.45 ± 1.71) *Between-group comparisons* ↓ PWC170 in IHHE compared to control group (pre-test: 170.8 ± 44.8 W vs. 204.2 ± 13.8 W; post-test: 191.9 ± 26.9 W vs. 278.0 ± 19.3 W) ↑ IRI in IHHE compared to control group (pre-test: 65.8 ± 3.6% vs. 50.8 4.1%; post-test: 54.8 ± 5.4% vs. 49.6 3.8%) ↑ CRI in IHHE compared to control group (pre-test: 50.0 ± 5.3% vs. 37.5 ± 4.9%) ↓ S_p_O_2_ during hypoxic test in IHHE compared to control group (pre-test: 77.9 ± 6.8% vs. 83.7 ± 9.0%) ↑ HR_max_ during hypoxic test in IHHE compared to control group (pre-test: 82.2 ± 14.6 beats/min vs. 79.7 ± 13.1 beats/min) ↓ R–R intervals in IHHE compared to control group (post-test: 890 ± 160 ms vs. 990 ± 180 ms) ↓ Standard deviation of R–R intervals in IHHE (54.0 ± 24.7 ms vs. 82.0 ± 24.8 ms) ↑ HR_rest_ in IHHE compared to control group (post-test: 67.1 ± 13.7 beats/min vs. 60.4 ± 4.6 beats/min) ↓ High frequency in IHHE compared to control group (pre-test: 277 ± 188 ms vs. 1100 ± 344 ms^2^; post-test: 624 ± 468 ms^2^ vs. 1167 ± 501 ms^2^) ↑ Low frequency to high frequency index in IHHE compared to control group (pre-test: 8.01 ± 7.51 vs. 2.2 ± 1.0)
Dudnik et al. [109]	*Physical functions* Cardiopulmonary exercise test *Cardiovascular hemodynamic parameters* Resting heart rate Resting systolic and diastolic blood pressure *Blood markers* Red blood cells count, white blood cell count, platelets, haemoglobin concentration, reticulocytes	*Within-group comparisons (pre-test vs. post-test)* ↑ VO_2peak_ in IHHE (13.9 ± 2.5 ml-O_2_/min/kg vs. 19.9 ± 6.1 ml-O_2_/min/kg) *Between-group comparisons* ↑ Reticulocytes in IHHE compared to sham IHHE (post-test: 1.1 ± 0.5% vs. 0.6 ± 0.3%) *Interaction effects (group* *×* *time)* ↓ Diastolic blood pressure in IHHE compared to sham IHHE (pre-test: 82.1 ± 11.1 mmHg vs. 77.9 ± 9.7 mmHg; post-test: 74.7 ± 8.9 mmHg vs. 82.0 ± 9.3 mmHg)
Glazachev et al. [110]	*Physical functions* Cardiopulmonary exercise test *Blood markers* Total cholesterol, high and low-density lipoprotein, triglycerides, and glucose Quality of life Medical Outcome Study 36-item Short Form Health Survey (MOS SF-36) Seattle Angina Questionnaire (SAQ)	*Within-group comparisons (pre-test vs. post-test vs. 1-month follow-up)* ↑ Time to exhaustion in cardiopulmonary exercise test (modified Bruce protocol) in IHHE (303 ± 147 s vs. 362 ± 124 s^a^ vs. 342 ± 113 s) ↑ Metabolic equivalent in IHHE (3.5 ± 1.2 vs. 39.1 ± 1.0 s vs. 4.2 ± 1.2 s) ↑ VO_2_ at anaerobic threshold in IHHE (11.5 ± 1.3 ml-O_2_/min/kg vs. 13.8 ± 2.0 ml-O_2_/min/kg^a^ vs. 13.8 ± 0.3 ml-O_2_/min/kg^b^) ↑ MOS SF-36 physical functioning subscale in IHHE (84.2 ± 13.0 vs. 55.7 ± 12.0^a^ vs. 51.7 ± 14.0) ↑ MOS SF-36 role physical subscale in IHHE (47.0 ± 17.8 vs. 61.7 ± 18.8^a^ vs. 55.8 ± 19.0) ↑ MOS SF-36 body pain subscale in IHHE (22.0 ± 39.4 vs. 48.5 ± 43.7 vs. 58.8 ± 39.0^b^) ↑ MOS SF-36 vitality subscale in IHHE *Between-group comparisons* ↑ MOS SF-36 physical functioning subscale in IHHE compared to sham IHHE (post-test: 61.7 ± 18.8 vs. 47.5 ± 11.9) ↑ MOS SF-36 body pain subscale in IHHE compared to sham IHHE (post-test: 48.5 ± 43.7 vs. 27.3 ± 8.9) ↑ metabolic equivalent in IHHE compared to sham IHHE (post-test: 3.5 ± 0.9 vs. 3.8 ± 1.0) ↑ VO_2peak_ in IHHE compared to sham IHHE (post-test: 16.9 ± 1.4 ml-O_2_/min/kg vs. 12.0 ± 6.3 ml-O_2_/min/kg)
Serebrovska et al. [66]	*Blood markers* Total cholesterol, high- and low-density lipoprotein, and triglycerides Fasting glucose level and 2 h post-oral glucose tolerance test glucose level *Hypoxia test (20 min at F*_*i*_*O*_*2*_ = *0.10)* Oxygen saturation (S_p_O_2_) Maximal heart rate (HR_max_)	*Within-group comparisons (pre-test vs. post-test vs. 1-month follow-up)* ↑ Minimum S_p_O_2_ during hypoxic test in IHHE (79.4 ± 3.8% vs. 81.5 ± 3.9^a^ % vs N.R.^b^) ↓ Fasting glucose in IHHE and IHE (IHHE: 6.3 ± 0.5 mmol/L vs. 5.8 ± 0.7 mmol/L^a^ vs. 5.3 ± 0.8 mmol/L^b^; IHE: 6.5 ± 0.4 mmol/L vs. 5.4 ± 0.5 mmol/L^a^ vs. 5.1 ± 0.6 mmol/L^b^) ↓ 2-h post-oral glucose tolerance test glucose level in IHHE and IHE (IHHE: 7.9 ± 0.9 mmol/L vs. 6.8 ± 1.0 mmol/L^a^ vs. 6.4 ± 1.3 mmol/L^b^; IHE: 8.3 ± 1.0 mmol/L vs. 7.0 ± 1.9 mmol/L^a^ vs. 6.4 ± 1.1 mmol/L^b^) ↓ Total cholesterol in IHHE and IHE (IHHE: 6.3 ± 1.1 mmol/L vs. 5.7 ± 1.0 mmol/L^a^ vs. 6.1 ± 1.3 mmol/L; IHE: 6.2 ± 1.2 mmol/L vs. 5.3 ± 0.9 mmol/L^a^ vs. 5.8 ± 1.2 mmol/L) ↓ Low-density lipoprotein cholesteral in IHHE and IHE (IHHE: 4.2 ± 1.3 mmol/L vs. 3.5 ± 1.0 mmol/L^a^ vs. 3.5 ± 1.3 mmol/L^b^; IHE: 4.0 ± 1.3 mmol/L vs. 3.3 ± 1.0 mmol/L^a^ vs. 3.4 ± 1.0 mmol/L) *Between-group comparisons* ↓ Fasting glucose in IHHE compared to sham IHHE (1-month follow-up: 5.3 ± 0.8 mmol/L; vs 6.1 ± 0.8 mmol/L) ↓ Fasting glucose in IHE compared to sham IHHE (post-test: 5.4 ± 0.5 mmol/L vs. 6.12 ± 0.8 mmol/L; 1-month follow-up: 5.1 ± 0.6 mmol/L vs. 6.1 ± 0.8 mmol/L) ↓ 2-h post-oral glucose tolerance test glucose level in IHHE compared to sham IHHE (post-test: 6.8 ± 1.0 mmol/L vs. 8.3 ± 1.1 mmol/L; 1-month follow-up: 6.4 ± 1.3 mmol/L vs. 8.2 ± 1.2 mmol/L) ↓ 2-h post-oral glucose tolerance test glucose level in IHE compared to sham IHHE (post-test: 7.0 ± 1.9 mmol/L vs. 8.3 ± 1.1 mmol/L; 1-month follow-up: 6.4 ± 1.1 mmol/L vs. 8.2 ± 1.2 mmol/L) ↓ Total cholesterol in IHE compared to sham IHHE (1-month follow-up: 5.3 ± 0.9 mmol/L vs. 6.2 ± 0.9 mmol/L)
Serebrovska et al. [111]	*Cognitive functions* Montreal Cognitive Assessment Test (MoCA) Long latency cognitive event-related potential (P300, N200) *Blood markers* Amyloid-β and amyloid precursor protein (APP130, APP110, and APP110/APP130 ratio) Beta-site amyloid precursor protein cleaving enzyme 1 (BACE1) Stimulated neutrophil extracellular traps formation in peripheral blood (NET_st_)	*Within-group comparisons (pre-test vs. post-test vs. 1-month follow-up)* ↑ MoCA test score in IHHE (19.6 ± 1.6% vs. 22.1 ± 1.7%^a^ vs. 21.3 ± 1.6%) ↑ APP130 in IHHE (0.4 ± 0.1 r.U. vs. 0.7 ± 0.1 r.U.^a^ vs. 0.6 ± 0.1 r.U.^b^) ↑ APP110 in IHHE (0.6 ± 0.1 r.U. vs. 0.7 ± 0.1 r.U.^a^ vs. 0.8 ± 0.1 r.U.^b^) ↑ APP-ratio in IHHE (0.7 ± 0.1 vs. 0.9 ± 0.1^a^ vs. 0.8 ± 0.1) ↓ Amyloid-β in IHHE (2.6 ± 0.3 r.U. vs. 2.2 ± 0.4 r.U.^a^ vs. 2.1 ± 0.4 r.U.^b^) ↓ NET_st_ in IHHE (12.7 ± 6.2% vs. 8.8 ± 3.3% vs. 6.1 ± 3.5%^b^) ↓ NET_ns_ in IHHE (9.5 ± 2.1% vs. 4.5 ± 1.1%^a^ vs. 4.2 ± 1.3%^b^) ↓ BACE1 in IHHE (85.3 ± 55.6 r.U. vs. 36.8 ± 34.6 r.U.^a^ vs. 45.6 ± 32.8 r.U.) *Between-group comparisons* ↑ APP130 in IHHE compared to sham IHHE (post-test: 0.7 ± 0.1 r.U. vs. 0.4 ± 0.1 r.U.; 1-month follow-up: 0.6 ± 0.1 r.U. vs. 0.4 ± 0.1 r.U.) ↑ APP110 in IHHE compared to sham IHHE (post-test: 0.7 ± 0.1 r.U. vs. 0.5 ± 0.1 r.U.; 1-month follow-up: 0.8 ± 0.1 r.U. vs. 0.5 ± 0.1 r.U.) ↑ APP-ratio in IHHE compared to sham IHHE (post-test: 0.9 ± 0.1 vs. 0.8 ± 0.1) ↑ Amyloid-β in IHHE compared to sham IHHE (post-test: 2.2 ± 0.4 r.U. vs. 2.8 ± 0.4 r.U.; 1-month follow-up: 2.1 ± 0.4 r.U. vs. 2.8 ± 0.2 r.U.) ↓ NET_st_ in IHHE (1-month follow-up: 6.1 ± 3.5% vs. 11.2 ± 3.6%) ↓ NET_ns_ in IHHE (post-test: 4.5 ± 1.1% vs. 9.22 ± 3.9%; 1-month follow-up: 4.2 ± 1.3% vs. 8.25 ± 2.0%) ↓ BACE1 in IHHE (85.3 ± 55.6 r.U. vs. 36.8 ± 34.6 r.U.^a^ vs. 45.6 ± 32.8 r.U.)
Bestavashvili et al. [112]	*Anthropometric parameters* Body mass index (BMI) Waist circumference Hip circumference *Blood markers* Total cholesterol, high- and low-density lipoprotein, and triglycerides Alanine aminotransferase (ALT) Aspartate aminotransferase (AST) Galectin-3 Nitric oxide synthase 2 (NOS2) Heat shock proteins (Hsp70) Transforming growth factor beta-1 (TGF beta-1) Heart-type fatty acid binding protein (H-FABP) High-sensitive C-reactive protein (CRP-hs) *N*-Terminal pro-hormone of brain natriuretic peptide (NTproBNP)	*Within-group comparisons (pre-test vs. post-test)* ↓ BMI in IHHE (34.2 ± 5.2 kg/m^2^ vs. 33.3 ± 5.2 kg/m^2^) ↓ Waist circumference in IHHE (116.2 ± 11.2 cm vs. 111.0 ± 10.6 cm) ↓ Hip circumference in IHHE (114.1 ± 9.4 cm vs. 110.3 ± 9.4 cm) ↑ Total cholesterol in sham IHHE (4.8 ± 1.2 mmol/L vs. 5.1 ± 1.1 mmol/L) ↓ ALT in IHHE (37.3 ± 26.1 u/L vs. 29.0 ± 15.3 u/L) ↓ HSP70 in IHHE (0.963 ± 0.316 ng/mL vs. 0.865 ± 0.334 ng/mL) ↓ CRP-hs in IHHE (3.608 ± 3.448 mg/L vs. 2.237 ± 1.527 mg/L) ↓ NTproBNP in IHHE (27.5 ± 45.1 pmol/L vs. 20.4 ± 34.2 pmol/L) *Between-group comparisons* ↓ ALT in IHHE compared to sham IHHE (post-test: 29.0 ± 15.3 u/L vs. 36.2 ± 21.5 u/L) ↓ NTproBNP in IHHE compared to sham IHHE (post-test: 20.4 ± 34.2 pmol/L vs. 34.9 ± 62.1 pmol/L) ↓ Δ-BMI in IHHE compared to sham IHHE (-0.9 ± 0.5 vs. 0.3 ± 0.6) ↓ *Δ*-Waist circumference in IHHE compared to sham IHHE (− 5.2 ± 2.4 vs. 0.7 ± 1.8) ↓ *Δ*-Hip circumference in IHHE compared to sham IHHE (− 3.8 ± 1.7 vs. 3.4 ± 1.0) ↓ *Δ*-Total cholesterol in IHHE compared to sham IHHE (− 0.8 ± 0.8 vs. 0.3 ± 0.1) ↓ *Δ*-Triglyceride in IHHE compared to sham IHHE (− 0.3 ± 0.4 vs. 0.1 ± 0.5) ↓ *Δ*-Low-density lipoprotein in IHHE compared to sham IHHE (− 0.8 ± 0.7 vs. 0.3 ± 0.8) ↓ *Δ*-ALT in IHHE compared to sham IHHE (− 8.3 ± 14.6 vs. 5.4 ± 9.2) ↓ *Δ*-AST in IHHE compared to sham IHHE (− 4.5 ± 12.1 vs. 3.2 ± 6.3) ↓ *Δ*-NTproBNP in IHHE compared to sham IHHE (− 7.1 ± 13.6 vs. 9.0 ± 18.0)

*ALT* Alanine aminotransferase, *APP* amyloid precursor protein, *AST* Aspartate aminotransferase, *BACE1* beta-site amyloid precursor protein cleaving enzyme 1, *BMI* body mass index, *CDT* Clock-drawing Test, *CRI* chronotropic reserve index, *CRP-hs* High-sensitive C-reactive protein *DemTect* Dementia Detection Test, *F*_*i*_*O*_*2*_ fraction of inspired oxygen, *H-FABP* Heart-type fatty acid binding protein, *Hsp70* Heat shock proteins, *HR*_*max*_ maximum heart rate, *HR*_*rest*_ resting heart rate, *IRI* inotropic reserve index, *IHE* intermittent hypoxic exposure, *IHHE* intermittent hypoxia–hyperoxia exposure, *IQR* interquartile range, *MoCA* Montreal-Cognitive-Assessment, *MOS SF-36* Medical Outcome Study 36-item Short Form Health Survey, *NET*_*st*_ stimulated neutrophil extracellular traps formation, *NET*_*ns*_ not stimulated neutrophil extracellular traps formation, *NOS2* Nitric oxide synthase 2, *N.R*. not reported, *NTproBNP* N-terminal pro-hormone of brain natriuretic peptide, *PWC130* physical work capacity at a heart rate of 130 beats min^−1^, *r.U.* relative units, *SAQ* Seattle Angina Questionnaire, *TGF beta-1* Transforming growth factor beat-1, *VO*_*2peak*_ peak oxygen uptake, *6MWT* Six-minute Walk Test

^a^*p* values < .05 for differences between pre-test and post-test (time effect)

^b^*p* values < .05 for differences between pre-test and 1-month follow-up (time effect)

^c^*p* values < .05 for differences between post-test and 1-month follow-up (time effect)

#### Physical Performance

Five of the eight included studies measured physical performance outcomes [106–110]. In three of these studies, a cardiopulmonary exercise test was performed before and after 3 [110] or 5 weeks [107, 109] of the IHHE intervention. In two studies, exercise tolerance (i.e. time until exhaustion while performing the Bruce or modified Bruce protocol) was increased at the end of the intervention [110] or 1-month follow-up [107] in patients who performed IHHE but not in those who performed sham IHHE. Significant improvements in peak oxygen consumption have been observed in older patients with coronary artery disease (+ 12.6%; pre: 14.3 ± 4.2 ml-O_2_/min/kg; post: 16.1 ± 4.2 ml-O_2_/min/kg) [107] and cardiology outpatients (+ 43.2%; pre: 13.9 ± 2.5 ml-O_2_/min/kg; post: 19.9 ± 6.1 ml-O_2_/min/kg) [109] after 5 weeks of IHHE when compared to baseline. In the study by Glazachev et al. [110], peak oxygen consumption (+ 26.1%; pre: 13.4 ± 2.5 ml-O_2_/min/kg; post: 16.9 ± 1.4 ml-O_2_/min/kg) and oxygen uptake at the first ventilatory threshold (+ 11.3%; pre: 11.5 ± 1.3 ml-O_2_/min/kg; post: 13.8 ± 2.0 ml-O_2_/min/kg) were increased in older patients with coronary artery disease who conducted 3 weeks of IHHE. Furthermore, the increase in peak oxygen consumption was higher in the IHHE group compared to the patients who had performed sham IHHE. Susta et al. [108] have found that the physical work capacity of young athletes with overtraining syndrome (i.e. the power at a heart rate of 170 beats/min, PWC 170) was improved after 4 weeks of IHHE which was performed 1.5–2 h after low-intensity aerobic exercise (two bouts of 30 min running at 50% of maximum oxygen uptake, with 10 min rest between bouts). In one study, older geriatric patients performed the six-minute walk test prior to and after 5–6 weeks of real or sham IHHE combined with a multimodal training program [106]. The improvement in the six-minute walk distance at the end of the intervention was higher in patients who completed the IHHE in combination with the multimodal training program than in patients who received sham IHHE plus multimodal training [106].

#### Cognitive Performance

The effect of IHHE on cognitive performance was investigated by two studies [106, 111] using different populations. With regard to older patients undergoing a multimodal training program (2–3 times per week for 5–6 weeks, consisting of 30 min of physiotherapy, 60 min of occupational therapy, and 20 min of aerobic exercise) in a geriatric day care unit, the additional application of IHHE led to improvements in global cognitive functions (i.e. operationalized by Dementia Detection Test score and Clock Drawing Test score) when compared with older patients performing the same multimodal training program combined with sham IHHE [106]. In older people with mild cognitive impairments, but not healthy controls, global cognitive functions (i.e. Montreal Cognitive Assessment Test) increased after 3 weeks of IHHE, whereas sham IHHE did not lead to a change in cognitive test performance [111]. However, 3 weeks of IHHE had no effect on N200 and P300 latency in both older people with mild cognitive impairments and healthy older people [111]. In the same study, participants with mild cognitive impairment who performed a 3-week IHHE intervention, showed an increase of neuroprotective proteins (i.e. amyloid precursor proteins) and a decrease in circulating biomarkers of Alzheimer’s disease (i.e. amyloid-beta, neutrophil extracellular traps, and beta-site amyloid precursor protein cleaving enzyme 1) in the peripheral blood [111].

#### Haematological, Metabolic, and Haemodynamic Parameters

In three studies, changes in haematological parameters were evaluated after 4–5 weeks of IHHE in older patients with coronary artery disease [107] and cardiac comorbidities [109] as well as young athletes with overtraining syndrome [108]. Increases in reticulocytes were found in patients with coronary heart disease after 3 weeks of IHHE when compared with patients who conducted 8 weeks of the standard rehabilitation program and 3 weeks of sham IHHE [107]. However, two other studies did not observe such a change in patients with cardiac comorbidities [109] as well as young athletes with overtraining syndrome [108]. All three studies [107–109] that investigated IHHE-related changes in red blood cell count and haemoglobin concentration did not find evidence for a change in response to the intervention. In a comparable manner, IHHE also had no effect on haematocrit level [108] or white blood cell count and platelets [109].

The patients’ metabolic status was assessed in four studies [66, 107, 110, 112] and in one of these studies [107], investigating older patients with coronary arterial disease, a reduction in total cholesterol level was observed compared with patients who had performed a standard rehabilitation program and sham IHHE. In two studies investigating the effects of IHHE in older patients with coronary arterial disease [110] and prediabetes [66], a reduction in total cholesterol levels was observed after 3 weeks of IHHE, while total cholesterol levels remained unchanged in those patients who had conducted sham IHHE. In another study [112], no change in total cholesterol was observed in older patients with metabolic disease. Two studies including older patients with coronary arterial disease [107] or prediabetes [66], reported reductions in low-density lipoprotein cholesterol after 3 weeks of IHHE compared to baseline, whereas only one of these studies [107] reported a reduced low-density lipoprotein cholesterol level compared to a sham IHHE group. Only one study [110] noticed a tendency towards a decrease in low-density lipoprotein cholesterol in patients with coronary arterial disease. With regard to patients with metabolic syndrome, 3 weeks of IHHE did not result in a reduction in low-density lipoprotein cholesterol blood concentration [112]. High-density lipoprotein cholesterol was increased after 3 weeks of IHHE in patients with coronary arterial disease compared to baseline [110], whereas the levels remained unchanged in patients with prediabetes [66] and metabolic syndrome [112]. Moreover, in the study of Glazachev et al. [107], a reduction in the atherogenic index (i.e. [total cholesterol – high-density lipoprotein cholesterol] ÷ high-density lipoprotein cholesterol) was found in patients with coronary arterial disease who had conducted IHHE over 5 weeks compared to those who had performed 8 weeks of the standard rehabilitation program and 3 weeks of sham IHHE. Moreover, one study [110] reported a decrease in triglyceride levels compared to baseline in response to 3 weeks of IHHE, but three other studies [66, 107, 112] did not reveal such an effect. In patients with prediabetes, fasting blood glucose concentration was reduced at the 1-month follow-up assessment and 2 h post-oral glucose tolerance test glucose levels were decreased 1 day after and remained decreased 1 month after a 3-week IHHE intervention when compared with patients with prediabetes conducting sham IHHE [66]. Additionally, Bestavashvili et al. [112] reported a decrease in body mass index, waist and hip circumference, and inflammatory markers in patients with metabolic syndrome after 3 weeks of IHHE compared to a sham IHHE group.

Four studies [106–109] evaluated the effect of IHHE on haemodynamic indices. In two studies that measured the effect of IHHE on blood pressure and heart rate recorded at rest in geriatric patients [106] and cardiac outpatients [109], no changes in systolic and diastolic blood pressure as well as heart rate were observed. In one study [107], both resting systolic and diastolic blood pressure as well as heart rate were decreased after 5 weeks of IHHE in patients with coronary artery disease when compared with baseline. Susta et al. [108] reported that 4 weeks of IHHE plus low-intensity aerobic exercise (2 bouts of 30 min) improved the inotropic reserve index (i.e. [maximum systolic blood pressure − resting systolic blood pressure] ÷ resting systolic blood pressure) and the chronotropic reserve index (i.e. [maximum heart rate − resting heart rate] ÷ resting heart rate) in healthy athletes with overtraining syndrome. In addition, the parasympathetic drive was increased (i.e. high-frequency power of heart rate variability), while the sympathetic tone was decreased (i.e. low-frequency power of heart rate variability and low-frequency power high-frequency power ratio) after 4 weeks of IHHE. One study [107] found an increase in left ventricular ejection fraction after 5 weeks of IHHE in patients with coronary arterial disease when compared to baseline. However, left ventricular ejection fraction did not differ between patients who conducted 5 weeks of IHHE and patients who performed 8 weeks of the standard rehabilitation program and 3 weeks of sham IHHE [107].

## Discussion

In this systematic review, we included eight studies that have investigated the chronic effects of intermittent hypoxia–hyperoxia on physical and cognitive performance as well as haemodynamic, metabolic, or haematological parameters in humans. All of the reviewed studies [66, 106–112] have performed intermittent hypoxia–hyperoxia at rest (i.e. IHHE), with intervention durations ranging from 3 to 6 weeks. Two studies implemented a physical training program in addition to the IHHE intervention [106, 108]. The parameters that were most frequently assessed included changes in (1) physical performance [106–110], (2) haemodynamic parameters [106–109], and (3) parameters of the metabolic state [66, 107, 110, 112]. Two of the reviewed studies [106, 111] have investigated the effects of IHHE on (4) cognitive performance and three studies [107–109] have investigated (5) haematological parameters. The results of some studies included in this systematic review seem conflicting and are difficult to compare due to the heterogeneity in study population and design.

### Effects of Intermittent Hypoxia–Hyperoxia Exposure on Physical Performance

The findings of our systematic review indicate that IHHE might have positive effects on physical performance in specific populations, such as in geriatric patients [106]. The improvements in physical performance could be explained by specific cardiovascular and muscular adaptations to IHHE, e.g. the regulation of inflammatory response, angiogenesis, improved glycolysis, glucose transport, and vasodilatation as well as mitochondrial functioning [4, 113]. Furthermore, it is assumed that physical exercise in hypoxia (e.g. aerobic exercise under continuous hypoxia) might be a great promise for successful geriatric rehabilitation by inducing lower mechanical stress compared to a similar training in normoxia (i.e. when the exercise intensity is equal and operationalized by a marker of internal load [e.g. heart rate]) [114]. An increased physical performance was also observed in young track and field athletes with overtraining syndrome, evidenced by an improved physical work capacity and balance of the autonomic nervous system (evaluated by changes in heart rate variability frequency measurements, i.e. low- and high-frequency power, and low- to high-frequency power ratio) [108]. The authors assumed that a recovered autonomic nervous system and an increased antioxidant capacity might partially explain these results [108]. However, this hypothesis remains speculative, since the authors did not measure the antioxidant status. Unfortunately, the control group consisted of healthy athletes who kept their training routine constant, and thus, the results could not be compared to a “real” control group in this pilot study, i.e. athletes with overtraining syndrome who have trained without an additional IHHE program or a sham IHHE. Therefore, no robust conclusions can be drawn concerning the synergistic effects of IHHE executed after low-intensity running in athletes with overtraining syndrome.

Three studies [107, 109, 110] imply that IHHE might be an effective intervention to increase peak oxygen consumption in patients with cardiovascular disease by 12.6–43.2% (~ 1.8–6.0 ml-O_2_/min/kg). In general, an increase of 3.5 ml-O_2_/min/kg is considered as the minimum important difference in cardiac rehabilitation [115]. Moreover, it was shown that an increase in peak oxygen consumption of 6% is associated with a 5% lower risk of all-cause mortality and morbidity in patients with heart failure [116]. Accordingly, the improvements in peak oxygen consumption observed after IHHE can be considered as clinically meaningful. A previous study [52] in which prolonged hypoxic exposures of 10 sessions of 3–4 h per session (F_i_O_2_ = 0.175–0.150) were used over a period of 22 days demonstrated a significant increase in peak oxygen consumption (~ 5%; pre: 13.5 ± 1.8 ml-O_2_/min/kg; post: 14.2 ± 1.9 ml-O_2_/min/kg) in patients with heart failure and reduced ejection fraction. However, this study included only 12 patients without a control group [52]. Another study by Burtscher et al. [49] included eight elderly, physically active males with New York Heart Association class I to II heart failure who were exposed to intermittent hypoxia (5 times per week, F_i_O_2_ = 0.14–0.10) and eight subjects of the same population who received an equivalent sham condition. The authors observed a significant increase in peak oxygen consumption from 2333 ± 586 ml-O_2_/min to 2475 ± 546 ml-O_2_/ml (~ 6%) after 3 weeks of intermittent hypoxic exposure without changes in the subjects who completed the sham condition [49]. However, the findings of a systematic review [26] suggest that passive hypoxia application can enhance exercise tolerance during submaximal exercise, but changes in maximal exercise capacity (e.g. peak oxygen consumption) were somewhat difficult to detect in healthy physically active individuals. This can be explained by the already high level of cardiorespiratory fitness when compared to patients with cardiovascular disease [49, 52, 117, 118]. Moreover, it should be noted that the participants in the studies [107, 109, 110] included in our systematic review were all of higher age (mean age from 63.9 to 68.2 years) and had cardiovascular diseases as well as low peak oxygen consumption values at baseline (13.4–14.3 ml-O_2_/min/kg). Thus, the observed effects of IHHE cannot be generalized to other populations such as healthy individuals.

### Effects of Intermittent Hypoxia–Hyperoxia Exposure on Cognitive Performance

The beneficial effects of a well-dosed application of intermittent hypoxia–normoxia or hypoxia–hyperoxia on neurocognitive health have recently been discussed by several authors [45, 67, 119, 120]. These reviews have summarized the evidence from research in various populations suggesting that IH can be applied as a therapeutic modality in order to preserve or enhance brain functions. Hence, the development and progression of age- or disease-dependent cognitive impairments such as mild cognitive impairments or dementia might be mitigated. For instance, investigations in animals and humans have found an improved cerebrovascular function (e.g. augmented cerebral blood flow due to enhanced endothelial-dependent vasodilatation and vascularisation) [121–123], reduced vascular risk factors (e.g. hypertension, hypercholesterolaemia, obesity) [50, 124, 125] and inflammation (e.g. due to the anti-inflammatory effect of erythropoietin [126, 127]), prevented neuronal degeneration [128], as well as stimulated neurogenesis and neuroregeneration [129, 130]. However, the results of our review suggest that clinical evidence on the neurocognitive effects of intermittent hypoxia–hyperoxia is currently limited. Bayer et al. [106] found that global cognitive performance only improved in those patients who underwent the combination of the multimodal training program and the IHHE. The authors concluded that the lack of improvements in cognitive performance in patients who conducted the multimodal training program in combination with sham IHHE might be explained by their low initial fitness level, which made it impossible to undergo training with an exercise intensity sufficient to induce measurable improvements in cognitive performance. Consequently, improvements in cognitive performance could be related to the effects of IHHE. However, more well-controlled studies are necessary to confirm these promising findings. Furthermore, Serebrovska et al. [111] reported a better cognitive performance one day after the last IHHE session, which was associated with a decrease in non-stimulated neurotrophic extracellular traps and amyloid-beta expression. Neurotrophic extracellular traps are released by neutrophils to initiate immune defence mechanisms [131] and increased formation of neurotrophic extracellular traps has been observed in patients with Alzheimer’s disease [132, 133]. In general, neurotrophic extracellular traps formation and amyloid-beta accumulation are suggested to play a role in the pathogenesis of Alzheimer’s disease, which offers an approach for the treatment of this disease [134, 135]. Given the finding that IHHE influenced the formation of neurotrophic extracellular traps and amyloid-beta expression, IHHE could be an interesting intervention for future studies aiming to prevent or decelerate cognitive decline. Furthermore, there is some evidence that intermittent hypoxic exposure alone [56] or in combination with resistance training [40] and prolonged hypoxic exposure in combination with endurance training [39] can improve cognitive performance in older patients with mild cognitive impairment or in healthy older people. Even if these results seem promising, further studies are urgently needed to investigate the effects of IHHE or IHHT on various domains of cognitive functions (e.g. inhibition, working memory, cognitive flexibility) because previous studies [106, 111] only investigated global cognitive functions with a total of 26 participants. Furthermore, future studies should investigate the neurobiological mechanisms driving these cognitive performance enhancements by assessing changes on the (1) molecular and cellular level (e.g. changes in brain-derived neurotrophic factor), (2) structural and functional level (e.g. using magnetic resonance imaging (MRI), functional MRI, functional near-infrared spectroscopy), and (3) socioemotional level (e.g. sleep quality) [136, 137].

### Effects of Intermittent Hypoxia–Hyperoxia Exposure on Haematological, Metabolic, and Haemodynamic Parameters

Three studies [107–109] focussed on haematological parameters without detecting changes in haemoglobin concentration. Comparable results have been observed in healthy older males receiving intermittent hypoxic exposure (5 min of hypoxia [F_i_O_2_ = 0.12] separated by 5 min of normoxia, 4 times a day, daily for 10 days) [138], whereas other studies reported an increase in haemoglobin concentration [49] (3–5 min of hypoxia [F_i_O_2_ = 0.15–0.12] separated by 3 min of normoxia, 5–6 times a day, 5 sessions per week for 3 weeks) or total haemoglobin mass (same protocol as [49]) [54]. However, a growing amount of evidence suggests that the hypoxia intensity, total duration of hypoxic exposure, and inter-session density (in particular the duration of the single hypoxic exposure per day) are crucial factors for haematological adaptations to hypoxia [139]. It can be assumed that haemoglobin mass increases on average by 1.1% per 100 h of hypoxia [140] and that the minimum duration to reach an acclimatization effect and trigger haematological responses would be at least 12 h per day with a hypoxia intensity corresponding to altitudes of 2500–3000 m (F_i_O_2_ = ~ 0.155–0.145) [141]. Furthermore, Wilber et al. [142] stated that lower hypoxia intensities corresponding to altitudes of 2000–2500 m (F_i_O_2_ = ~ 0.165–0.155) would require a daily hypoxic duration of more than 22 h to achieve haematological changes. In three of the reviewed studies [107–109], the total hypoxic duration and the single hypoxic exposure per day were considerably lower than these values (i.e. 22 h). Thus, it can be assumed that the hypoxic dose was not sufficient to increase erythropoiesis. As a consequence, the improvements in exercise capacity were likely due to non-haematological adaptations such as respiratory (e.g. increased ventilatory efficiency), cardiovascular (e.g. increased stroke volume), or muscular or metabolic (e.g. improved mitochondrial efficiency and muscle pH-regulation) adaptations [4, 113]. Although one study [107] has shown that IHHE was associated with an improved cardiac function (i.e. increased left ventricular ejection fraction), the underlying mechanisms for the improvements in exercise capacity in response to IHHE are still not fully clarified and should be further investigated in additional studies in more detail.

The individual blood lipid profile (e.g. total cholesterol, high-density lipoprotein cholesterol, low-density lipoprotein cholesterol, and triglyceride concentration), blood glucose level, and blood pressure are important indicators concerning the assessment and management of health-related risk factors. Among other factors, their purposeful modification (e.g. due to interventions) may have a great importance for the prevention of metabolic and cardiovascular diseases [143–145].

In theory, hypoxia could induce positive effects on blood lipid levels by the modification of transcriptional factors that are responsible for the regulation of appetite (e.g. acylated ghrelin) [125, 146] as well as the glucose and lipid metabolism (e.g. proliferator-activated receptor gamma coactivator 1-α) [147–149]. However, none of the included studies [66, 110, 112] provide evidence for a robust effect of IHHE on blood lipid levels, except one [107]. Total cholesterol was significantly reduced in three studies [66, 107, 110] and remained unchanged in one study [112]. However, only Glazachev et al. [107] have demonstrated significant differences between patients who underwent IHHE and patients who underwent sham IHHE. In the same study [107], the atherogenic index (i.e. [total cholesterol − high-density lipoprotein cholesterol] ÷ high-density lipoprotein cholesterol) was significantly reduced in patients conducting IHHE compared to those who performed a standard rehabilitation program and sham IHHE. High-density lipoprotein cholesterol was significantly increased and triglycerides were significantly decreased over time but without differences between groups (e.g. in the latter study by Glazachev et al. [110]), while IHHE had no influence on these parameters in other studies [66, 112]. With regard to low-density lipoprotein cholesterol, both time- and group-effects were only observed in an earlier study conducted by Glazachev et al. [107], which has shown a decrease in low-density lipoprotein cholesterol after performing IHHE. In a comparable manner, Tin’kov et al. [51] have demonstrated that 22 daily sessions of continuous hypoxic exposure (3 h per session, F_i_O_2_ = ~ 0.135) resulted in a significant decrease in total cholesterol, low-density lipoprotein cholesterol, and triglycerides, whereas high-density lipoprotein cholesterol was increased in male patients with coronary artery disease. However, the study did not contain a control group that was not exposed to hypoxia. In general, the findings of the studies that have investigated the effect of continuous hypoxic training on blood lipid levels are relatively heterogeneous [124, 150–152]. Hence, there is currently little evidence supporting a positive effect of IHHE on blood lipid profile. Thus, further research is needed to draw robust conclusions.

A previous in vitro study has shown an increased insulin-independent glucose uptake in overweight or obese humans after 7 consecutive days of intermittent hypoxic exposure (3 cycles of 2 h exposures to hypoxia [F_i_O_2_ = 0.15], interspersed with 1 h of normoxia) [153]. The authors concluded that intermittent hypoxic exposure led to increases in glucose uptake via adenosine monophosphate-activated protein kinase-dependent pathways primarily in the myotubes but not in adipocytes. Moreover, activation of the HIF-1α subunit led to the induction of several genes involved in the glucose metabolism such as glucose transporter 1 and phosphofructokinase [154, 155]. In particular, increasing evidence suggests that long- and short-time IH (passive or in combination with exercise) may improve glucose uptake and insulin sensitivity in patients with diabetes mellitus type 2, metabolic syndrome, and overweight or obese patients [42, 156]. Based on the evidence mentioned above, intermittent hypoxic exposure or training might be an efficient, non-pharmacological therapeutic strategy to improve glucose metabolism in metabolically compromised individuals. The results of the present review point in the same direction as such positive effects were also observed after IHHE. However, this evidence has to be regarded as preliminary because only two studies [66, 110] have investigated fasting blood glucose concentration before and after an IHHE intervention. Serebrovska et al. [66] have shown that fasting blood glucose and 2 h post-oral glucose tolerance test glucose concentrations in patients with prediabetes were reduced one day after a 3-week IHHE intervention (5 sessions per week). Furthermore, glucose levels were still reduced at the 1-month follow-up assessment and were significantly lower compared to a sham IHHE group. Although no significant group differences were observed between IHHE and intermittent hypoxic exposure, the authors concluded that IHHE is more advantageous due to the reduction in session duration resulting from shorter reoxygenation periods (3 min during IHHE and 5 min during intermittent hypoxic–normoxic exposure). However, the authors did not investigate the effect of intermittent hypoxic exposure with shorter normoxic periods (e.g. 3 min). Indeed, previous studies have shown that intermittent hypoxic exposure can be effective to improve physical performance (i.e. peak oxygen consumption [49] and peak power [54]) in patients with heart failure or chronic obstructive pulmonary disease and to reduce blood pressure in hypertensive patients [50] even with shorter normoxic reoxygenation periods of 3 min. Considering these deficits, further studies are required to examine the effects of IHHE and IHHT on glucose metabolism in metabolically compromised persons.

In addition to the effects on blood lipid and glucose concentration, the influences of IHHE on resting systolic and diastolic blood pressure were also investigated [106, 107, 109]. The prevalence and absolute burden of hypertension is rising worldwide [157] and represents one of the leading modifiable risk factors for cardiovascular diseases being indirectly involved in the development of, for instance, kidney diseases and dementia [157, 158]. There is rather solid evidence supporting the assumption that intermittent and continuous hypoxia at rest or in combination with exercise is generally effective to reduce blood pressure [49, 50, 138, 159] and positively influence vascular health [41]. The mechanisms associated with an antihypertensive effect of moderate hypoxia may include vascular adaptions (e.g. increased vascularisation and endothelium-dependent vasodilatation) as well as adaptations in the autonomic nervous system (e.g. reduced sympathetic activity) [159]. From a physiological point of view, acute exposure to hypoxia is associated with an increase in blood flow, which is accompanied by higher endothelial shear stress and thereby endothelium-dependent increase in nitric oxide [160]. It is well known that nitric oxide causes vasodilatation [161], which reduces total peripheral resistance and thus blood pressure. Moreover, the hypoxia-mediated factor HIF-1α is also associated with antihypertensive mechanisms due to the upregulation of transcriptional genes such as nitric oxide synthase [162] (i.e. vasodilatation) and vascular endothelial growth factor [17] (i.e. vascularisation). Three studies have found that IHHE can decrease systolic (− 2.9% to − 13.9%) and diastolic blood pressure (− 9.0% to 14.0%) [106, 107, 109], although the changes did not always reach statistical significance [106, 109]. With regard to studies using intermittent hypoxia–normoxia, Lyamina et al. [50] exposed young males with stage I hypertension to 20 consecutive days of intermittent hypoxic exposure (4–10 cycles per session, 3 min of hypoxia [F_i_O_2_ = 0.10] interspersed by 3 min of normoxia) and found a decrease of 22 mmHg in systolic and 16.6 mmHg in diastolic blood pressure. In a more recent study, Muangritdech et al. [163] reported significant reductions in systolic blood pressure (-11.0 ± 9.7 mmHg) after 6 weeks of intermittent hypoxic interval training (2 sessions per week, 8 cycles per session, 3 min of hypoxia [S_p_O_2_ = 90.8 ± 2.31% to 87.7 ± 1.89%] interspersed by 3 min of normoxia combined with continuous treadmill walking at 35–50% of the participants’ individual heart rate reserve). Moreover, Serebrovska et al. [159] reported decreases of 10–30 mmHg in systolic and 10–15 mmHg in diastolic blood pressure in patients with stage I to II hypertension after intermittent or prolonged hypoxic exposure in their review. Recent meta-analyses have shown that every reduction of 10 mmHg in systolic or 5 mmHg in diastolic blood pressure reduced the risk of major cardiovascular events by 20%, the genesis of cardiovascular diseases by 17–40%, and all-cause mortality by 13% [164, 165]. Indeed, a decrease of even 2 mmHg in systolic blood pressure would involve a 10% lower stroke mortality and about 7% lower mortality for cardiovascular heart diseases or other vascular causes in middle age [166]. Given the evidence that IHHE can trigger a reduction in systolic and diastolic blood pressure in older patients with and without cardiovascular diseases, IHHE can be considered as a promising therapeutic strategy to reduce systemic blood pressure in this population. Therefore, the hypotensive effect of IHHE is practically relevant to prevent the genesis or exacerbation of cardiovascular diseases and ensure a healthy life.

### Hypoxia Dose

In general, the acute and chronic responses to hypoxia are complex and could be either adaptive/beneficial or maladaptive/pathological depending, among other factors, on the hypoxic dose. The hypoxic dose can be adjusted by modulating various variables (Table 4) including the (1) intensity of hypoxia (hyperoxia), (2) duration of a single hypoxic period as well as (3) intra-session frequency, and (4) intra-session density [25, 76]. Indeed, the variables mentioned above are relevant factors for the acute effects in response to a single IH session. In order to provide a more detailed explanation of the effects of the hypoxic dose on chronic adaptations, we suggest consideration of three additional variables (Table 4) which are relevant in an IH training program (i.e. when IH sessions are conducted regularly in a planned, structured, and purposive manner with the objective to increase or maintain at least one fitness or health dimension). These variables include the (5) inter-session frequency, (6) inter-session density, and (7) duration over which the IH intervention is carried out. In a review, Navarrete-Opazo and Mitchell [25] concluded that the intensity and the intra-session frequency of the hypoxic stimulus are the most important variables with regard to the acute and chronic responses to IH. Accordingly, the authors recommended “low-dose” IH protocols with an intensity of F_i_O_2_ = 0.09–0.16 and intra-session frequency of 3–15 cycles per session or day to achieve positive effects on multiple structures such as the cardiovascular, respiratory, musculoskeletal, neuronal, and immune systems [25]. Although the recommendations refer to IH sessions with normoxic reoxygenation periods, the studies in our review generally point in a comparable direction. Based on the current literature, we propose general recommendations for planning IHHE interventions in Table 4. However, given the evidence showing that acute and chronic responses to hypoxia are complex, specific, and inter-individual [167, 168], we also advocate for the conduction of additional high-quality studies investigating the acute and chronic dose–response relationship of IHHE and IHHT. Furthermore, we suggest that the administration of hypoxia and hyperoxia requires an individually tailored approach [77].

**Table 4:** Overview of the general variables determining the hypoxic dose and preliminary synopsis for the application of intermittent hypoxic–hyperoxic exposure (IHHE) interventions

Variable and description	IHHE protocol^a^
*Main variables relevant for a single IHHE session (acute effects)*	
Intensity of hypoxia	
Level of hypoxemia, typically reported as oxygen saturation of the blood (S_p_O_2_, internal intensity) or fraction of inspired oxygen (F_i_O_2_, external intensity)	F_i_O_2_ = 0.10–0.12
Intensity of hyperoxia	
Fraction of inspired oxygen (F_i_O_2_, external intensity) during hyperoxic periods	F_i_O_2_ = 0.30–0.40
Duration of a single hypoxic period	
Time spent in hypoxia before the onset of reoxygenation period (i.e. onset of normoxia or hyperoxia period)	2–6 min
Intra-session frequency	
Number of hypoxic periods that are interspersed with hyperoxic or normoxic periods (cycle) within a single session or day	4–8 cycles
Intra-session density	
Distribution of hypoxic periods across a distinct time interval with regard to reoxygenation time (i.e. duration of normoxia or hyperoxia period) within a single session or day	1–4 min
*Main variables relevant for a IHHE training program (chronic effects)*	
Inter-session frequency	
The number of IHHE sessions across a distinct time interval	3–5 sessions per week
Inter-session density	
Distribution of IHHE sessions across a distinct time interval with regard to recovery time in-between the IHHE sessions	Every second day until daily for 5 days interspersed with 2 days rest
Duration	
IHHE intervention duration	≥ 3 weeks

^a^Please note that the displayed variables were frequently reported in the reviewed studies and can serve as starting point for future investigations. However, currently no specific recommendations concerning the dose being most suitable for a distinct population can be provided since there is not enough evidence in the literature allowing us to draw robust and reliable conclusions in this direction

With regard to the intensity of the hypoxic stimulus, it is crucial to differentiate between internal (e.g. individuals’ S_p_O_2_) and external intensity (e.g. F_i_O_2_). Reductions in S_p_O_2_ at a fixed F_i_O_2_ vary widely within and between individuals due to different compensatory processes especially with increasing hypoxia intensity [168]. Knowing that internal intensity but not the external intensity determines the individuals’ physiological stress, it is suggested that the administration of hypoxia requires an individually tailored approach [77, 169]. To deal with this issue, the majority of the studies included in this systematic review [106–110, 112] performed a hypoxic test in order to examine patients’ individual response to hypoxia (i.e. changes in S_p_O_2_ and heart rate). Subsequently, the hypoxia intensity and intra-session density of the IHHE program was individually tailored based on the results of this hypoxia test [170]. Furthermore, to control and adjust the hypoxia intensity and duration during the IHHE session, the patients’ heart rate and S_p_O_2_ were monitored and the IHHE session was controlled via biofeedback (i.e. when reaching the individual minimum of S_p_O_2_, the hypoxic switched to the hyperoxic period until the initial S_p_O_2_ was reached).

Of note, the main difference of IHHE or IHHT compared to other IH methods is the replacement of normoxic periods by hyperoxic periods. This modification is hypothesized to up-regulate specific transcription factors [58, 59, 67], which can, in turn, cause adverse (e.g. cell damage) or beneficial (e.g. redox signalling) effects depending on the dose of the stimuli [25, 76]. In this regard, it has been shown that chronic exposure to hyperoxia can increase oxidative stress, which may have a negative impact on normal cellular mechanisms [171]. Thus, hyperoxia should be carefully administered even in IHHE or IHHT. With regard to the included studies, no adverse effects being directly attributable to the hyperoxic periods were reported. However, further research is necessary to better understand the biological consequences and possible health risks (e.g. for specific populations such as patients with chronic obstructive pulmonary disease) of the replacement of normoxic periods with hyperoxic periods. Unfortunately, due to the low number of studies and the heterogeneity in study population and design, a more detailed sub-analysis regarding the influence of the hypoxia and hyperoxia dose was not possible.

## Limitations

The first limitation is that four studies were excluded because they were not written in English. These studies were published between 2010 and 2017 and have investigated the effect of IHHE on different performance- and health-related outcomes in patients with metabolic and cardiovascular diseases. Unfortunately, the full-texts of all of these studies were published in Russian and could not be completely analysed as none of the authors of this systematic review understands Russian sufficiently well. Secondly, according to our quality assessment (modified Downs and Black checklist [75]), the majority of studies were classified as moderate quality. Thus, our findings should be viewed with respect to this limitation. In this context, a major point of concern is the insufficient justification of the sample size since only the study by Dudnik et al. [109] calculated and reported the sample size and effects size measures. The sample size calculation is a critical element of interventional studies as most of these studies aim to determine the effect (size) of different intervention approaches on a primary outcome parameter [172]. Therefore, the sample size calculation is a crucial part of the study planning being related to ethical, medical, and statistical considerations. In line with established recommendations [173], researchers are advised to pay more attention to an appropriate sample size calculation to improve the quality and transparency of their studies which, in turn, can enhance the robustness and trustworthiness of their findings. Furthermore, all of the reviewed studies lacked important methodological descriptions concerning Items 15, 19, 22, 24, and 28 of the modified Downs and Black checklist (see Risk of bias assessment [75]). In particular, the lack of reporting of the patients' compliance with the intervention is worth mentioning, given that reduced or marked inter-group differences in the patients’ compliance could have biased the effects of IHHE on performance- and health-related outcomes. In addition, the intervention was primarily applied in older patients with various diseases such as cognitive, cardiovascular, or metabolic disorders. Finally, the studies showed a strong heterogeneity with regard to their primary outcome parameters making a meta-analytical approach not possible.

## Conclusion and Perspective

Despite a somewhat limited number of studies included in our qualitative analysis, the current systematic review provides first hints that IHHE can be a non-pharmacological intervention strategy for improving peak oxygen consumption, exercise tolerance, and cognitive performance as well as reducing cardiometabolic risk factors (particularly blood glucose level, systolic and diastolic blood pressure) in older patients with cardiovascular and metabolic diseases or cognitive impairment. Importantly, although the results appear promising, more high-quality randomized controlled trials with a detailed description of the hypoxia dose and population (i.e. specific disease phenotype) are warranted before robust conclusions for the use of IHHE in therapy or clinical practice can be drawn. The evidence concerning the effects of IHHE on total cholesterol, high- and low-density lipoprotein cholesterol, and triglyceride blood level as well as erythropoiesis and haemoglobin mass is still inconclusive. Moreover, there is no evidence that replacing normoxic periods with hyperoxic periods enhances hypoxia-related adaptations in humans. This is mainly due to the fact that only one study directly compared the effect of IHHE and intermittent hypoxic exposure on blood glucose and lipoprotein cholesterol level in older patients with prediabetes.

Given the relatively low number of studies investigating the chronic effects of IHHE on performance- and health-related outcomes, there are some important aspects that should be addressed in future studies. These include the direct comparison of the effectiveness of IHHE or IHHT and hypoxic–normoxic exposure or training on, for example, changes in physical performance (e.g. exercise tolerance), cognitive performance (e.g. working memory), or cardiometabolic risk factors (e.g. systolic and diastolic blood pressure). Moreover, the cellular and molecular changes (e.g. nitric oxide, erythropoietin, HIF-1α) driving the adaptations to IHHE or IHHT should be examined. Furthermore, to better individualize IHHE or IHHT interventions, the optimal combination of variables that determine the dose–response relationship needs to be investigated with respect to physiological and structural adaptations as well as their importance for physical and cognitive performance improvements. These variables include the intensity of hypoxia and hyperoxia, the duration of a single hypoxic period, the intra-session frequency (i.e. the number of cycles), the intra-session density (i.e. duration of a single hyperoxic period), the inter-session frequency, the inter-session density, and the duration over which the IHHE or IHHT intervention is carried out (see Table 4). Finally, there are no studies available that have investigated the chronic effects of IHHT on performance- and health-related outcomes in humans. To address this gap, future studies are needed that investigate the combination of intermittent exposures to hypoxic and hyperoxic periods with different types of exercise, such as intermittent or continuous aerobic exercise or resistance exercise, to elucidate whether synergistic effects occur. In particular, it should be noted that the functional and structural adaptations in response to acute or chronic IHHT are not necessarily the same as those that occur during exercise in continuous hypoxia or intermittent hypoxia–normoxia. Therefore, current recommendations for exercise and training in hypoxic conditions should be re-evaluated for IHHT. As a consequence, it could be necessary to introduce specific recommendations for IHHT.

## Data Availability

The data within this systematic review are secondary data and are available through the relevant articles referenced throughout.

## Abbreviations

EPO: Erythropoietin
F_i_O_2_: Oxygen fraction in the inspired air
HIF: Hypoxia-inducible factors
HIF-1α: α-Subunit of HIF
HSP: Heat shock proteins
IH: Intermittent hypoxia
IHH: Intermittent hypoxia–hyperoxia
IHHE: Intermittent hypoxic–hyperoxic exposure
IHHT: Intermittent hypoxic–hyperoxic training
Keap1: Kelch-like ECH-associated protein 1
NF-κB: Nuclear factor kappa B
Nrf2: Nuclear factor erythroid 2-related factor
2PHD: Prolyl hydroxylase
P_i_O_2_: Oxygen partial pressure in the atmosphere
PRISMA: Preferred reporting items for systematic reviews and meta-analyses
ROS: Reactive oxygen species
S_a_O_2_: Arterial oxygen saturation
SpO_2_: Peripheral oxygen saturation
VEGF: Vascular endothelial growth factor
